# Multifunctional Janus-Structured Polytetrafluoroethylene-Carbon Nanotube-Fe_3_O_4_/MXene Membranes for Enhanced EMI Shielding and Thermal Management

**DOI:** 10.1007/s40820-025-01647-x

**Published:** 2025-02-06

**Authors:** Runze Shao, Guilong Wang, Jialong Chai, Jun Lin, Guoqun Zhao, Zhihui Zeng, Guizhen Wang

**Affiliations:** 1 Key Laboratory for Liquid-Solid Structural Evolution and Processing of Materials (Ministry of Education), Shandong University, Jinan, 250061 People’s Republic of China; 2 Center for Advanced Studies in Precision Instruments, Hainan University, Haikou, 570228 People’s Republic of China

**Keywords:** MXene, Polytetrafluoroethylene, Fe_3_O_4_, Janus-structured, EMI shielding, Thermal management, Multifunctional

## Abstract

Herein, a novel Janus-structured multifunctional membrane with integrated electromagnetic interference (EMI) shielding and personalized thermal management is fabricated using shear-induced in situ fibrillation and vacuum-assisted filtration. Interestingly, within the polytetrafluoroethylene (PTFE)-carbon nanotube (CNT)-Fe_3_O_4_ layer (FCFe), CNT nanofibers interweave with PTFE fibers to form a stable “silk-like” structure that effectively captures Fe_3_O_4_ particles. By incorporating a highly conductive MXene layer, the FCFe/MXene (FCFe/M) membrane exhibits excellent electrical/thermal conductivity, mechanical properties, and flame retardancy. Impressively, benefiting from the rational regulation of component proportions and the design of a Janus structure, the FCFe/M membrane with a thickness of only 84.9 µm delivers outstanding EMI shielding effectiveness of 44.56 dB in the X-band, with a normalized specific SE reaching 10,421.3 dB cm^2^ g^−1^, which is attributed to the “absorption-reflection-reabsorption” mechanism. Furthermore, the membrane demonstrates low-voltage-driven Joule heating and fast-response photothermal performance. Under the stimulation of a 3 V voltage and an optical power density of 320 mW cm^−2^, the surface temperatures of the FCFe/M membranes can reach up to 140.4 and 145.7 °C, respectively. In brief, the FCFe/M membrane with anti-electromagnetic radiation and temperature regulation is an attractive candidate for the next generation of wearable electronics, EMI compatibility, visual heating, thermotherapy, and military and aerospace applications.

**Supplementary Information:**

The online version contains supplementary material available at 10.1007/s40820-025-01647-x.

## Highlights

- The Janus-type multifunctional ultra-flexible polytetrafluoroethylene-carbon nanotube-Fe_3_O_4_/MXene (FCFe/M) membranes were fabricated via a shear-induced in situ fibrillation technique followed by vacuum-assisted filtration.
- Thanks to the strategic distribution of the MXene conductive reflection layer and the silk-like FCFe electromagnetic wave’s absorption layer, the membranes achieve robust electromagnetic interference shielding and effective antireflection through the absorption-reflection-reabsorption mechanism.
- The membranes exhibit exceptional thermal management performance, including efficient heat dissipation and electrothermal/photothermal conversion capabilities, further enhancing their promising potential for applications in flexible wearable technologies.

**Supplementary Information:**

The online version contains supplementary material available at 10.1007/s40820-025-01647-x.

## Graphical abstract

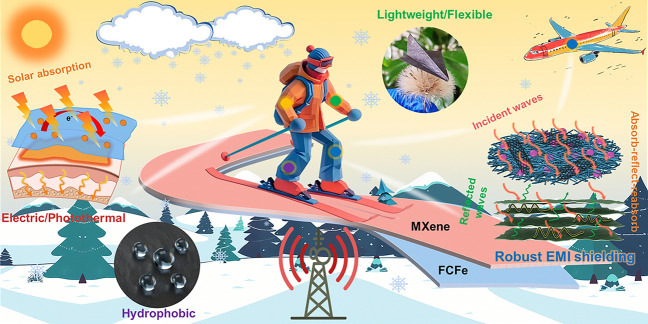

## Introduction

With the advent of next-generation communication technologies and the rapid evolution of portable electronic devices, flexible wearable electronics are increasingly becoming an integral part of our daily lives [1]. However, these devices inevitably generate electromagnetic (EM) radiation and interference during operation, which not only disrupts the normal functioning of surrounding electronic equipment, but also poses significant health risks to humans [2, 3]. While traditional metal-based shielding materials have been favored for their excellent electrical conductivity, their substantial weight, susceptibility to corrosion, processing difficulties, and lack of flexibility have increasingly made them less suitable for the evolving demands of modern society [4, 5]. Meanwhile, the significant impedance mismatch on their surface leads to a substantial portion of electromagnetic waves (EMWs) being reflected back into the surrounding environment, which not only ineffective in alleviating radiation leakage, but also susceptible to inducing undesirable secondary pollution [6, 7].

In recent years, conductive polymers and their composites (CPCs) with porous structures have attracted considerable attention owing to their lightweight, flexibility, robust corrosion resistance, and remarkable design versatility [8–10]. The combination of the continuous conductive network and the lightweight porous structure within CPCs effectively attenuates the propagation of EMWs through the conductive polymer [11, 12]. To date, a plethora of CPCs have been developed for electromagnetic interference (EMI) shielding, including carbon-based composites [13], graphene-based composites [14], and carbonaceous material foams [15]. Simultaneously, several strategies have been proposed to enhance the EMI shielding performance of CPCs with porous structure. One approach involves increasing the concentration of conductive fillers, but this method not only hinders effective integration between the fillers and the polymer matrix, but also exacerbates reflection effects, leading to additional EM pollution [16]. Another approach is to increase the thickness of CPCs, yet this method restricts its applicability in flexible wearable devices and portable electronic devices [17]. Consequently, there is a pressing need for a multifunctional and innovative design that integrates robust shielding and effective antireflection with thin and flexible characteristics.

A rational approach to resolve this paradox is to tailor the local conductivity of materials to fulfill varying requirements, by strategically positioning optimized compositions and architectures in appropriate regions, thereby integrating antireflective and shielding structures within a single asymmetric material [18]. Grounded in impedance matching theory, constructing multilayer materials featuring a continuous conductivity gradient offers the potential for near-perfect antireflective EMI shielding [19]. Despite this approach is conceptually straightforward, the intricate layer-by-layer fabrication process and the weak interlayer interactions substantially compromise the overall material performance [20, 21]. Therefore, developing a simpler and more feasible approach to fabricate asymmetric structures with superior performance remains a significant challenge. Recently, Janus-structured materials have garnered widespread attention due to their unique asymmetric structure and straightforward fabrication process [22, 23]. In comparison to conventional homogeneous materials, Janus structures possess distinctly different chemical compositions and microstructures on each side, enabling them to exhibit superior multifunctional performance through asymmetric synergistic effects and independent mechanisms [24, 25]. Particularly in the field of EMI shielding, designing one side of the material as an EMW reflective layer composed of high-conductivity materials, and the other side as an EMW absorbing layer based on CPCs can induce an “absorb-reflect-reabsorb” EMI shielding mechanism, which effectively shields EMWs while minimizing reflection effects [26].

Unlike the aforementioned concepts, the strategic selection of EM modulation components plays a crucial role in constructing conductivity-Janus structures and enhancing shielding performance. Due to their flexible assembly characteristics, emerging two-dimensional transition metal carbides and carbonitrides (MXene) have increasingly been utilized in the microstructural design of advanced EMI shielding materials [27]. Moreover, their outstanding metallic-like conductivity and distinctive layered architecture facilitate the formation of continuous and dense conductive networks during self-assembly, making them excellent candidates for conductive reflective layers in Janus structures [28, 29]. Notwithstanding unparalleled electrical properties, most assembled MXene films/papers are susceptible to mechanical deformation due to their inherent rigidity and brittleness, which constrains their use in flexible electronic applications [30]. Moreover, their hydrophilic nature and poor chemical stability further hinder their long-term performance in humid or harsh environments [18, 31]. Accordingly, developing a CPCs substrate with optimal flexibility and robust bonding with MXene is crucial for constructing Janus structures with enhanced structural stability and superior shielding performance.

PTFE is renowned for its exceptional mechanical strength, excellent chemical stability, and heat/cold resistance [32, 33]. Additionally, its inherent hydrophobicity and flexibility further broaden its applications in textiles, filtration systems, and electronic devices [34]. Therefore, employing PTFE-based CPCs as an EMW absorbing layer not only enhances the overall mechanical properties of Janus films, but also shields the conductive layer from external environmental damage, thereby extending the lifespan of electronic devices [35]. Nevertheless, given the robust solvent resistance of PTFE and its notably high melt viscosity, it is tough to be processed and modified PTFE-based CPCs via the traditional processing methods (freeze-drying, solution or melt spinning, phase separation, etc.) [36]. The difficulty is further compounded when attempting to fabricate PTFE-based composites that simultaneously achieve high filler content, excellent mechanical strength, and high porosity.

To address the aforementioned issues, we employed a straightforward and innovative shear-induced in situ fibrillation process to fabricate PTFE-CNT-Fe_3_O_4_ (FCFe) substrates. Impressively, the shear force during processing induces PTFE fibrillation, intertwining with CNT fibers to form a robust silk-like porous structure. This unique structure not only promotes the incorporation of Fe_3_O_4_ particles, boosting the composite films' magnetic loss capabilities, but also enhances EMW absorption through multiple internal reflections and scattering. Subsequently, the Janus PTFE-CNT-Fe_3_O_4_/MXene (FCFe/M) membrane was obtained through a simple vacuum-assisted filtration process. In terms of results, the FCFe/M membranes achieve efficient shielding and effective antireflection through the absorption-reflection-reabsorption mechanism. The Janus-structured membrane, with a thickness of just 84.9 µm, achieves a maximum EMI shielding effectiveness (SE) of up to 44.56 dB, and the normalized surface-specific SE (SSE, defined as the ratio of SE to the density and thickness of the shielding material) reaching an impressive 10,421.3 dB cm^2^ g^−1^. Additionally, owing to the inherent properties of the material and the anisotropy provided by the Janus structure, the FCFe/M membranes exhibit excellent mechanical properties, self-extinguishing characteristics, hydrophobicity, corrosion resistance, and electrothermal/photothermal conversion capabilities. This study presents a versatile strategy for developing EMI shielding membranes with robust shielding and effective antireflective properties. The resulting Janus FCFe/M composite membranes are anticipated to become strong candidates for future applications in EM radiation protection and next-generation flexible wearable technologies.

## Experimental Section

### Materials and Chemicals

PTFE powder (METABLEN A3800) was purchased from Mitsubishi Rayon Chemical, Japan. Single-walled carbon nanotubes (SWCNTs) with an outer diameter of 1.2–2.0 nm, known for their excellent electrical conductivity, were supplied by OCSiAl, Russia. Fe_3_O_4_ (200 nm) was purchased from Macklin Biochemical Technology Co., Ltd., Shanghai. Polylactic acid (PLA, Ingeo4032D), used as a lubricant during the in situ fiber formation process, was purchased from NatureWorks LLC, USA. The Ti_3_C_2_Al (400 mesh) and Celgard 3501 membrane (50 mm) were provided by Jilin Province 11 Technology Co., Ltd. Hydrochloric acid (HCl, 37 wt%), lithium fluoride (LiF), n-butyl alcohol, and dichloromethane (DCM) were purchased from Aladdin Biochemical Technology Co., Ltd., Shanghai.

### Preparation of the FCFe Membranes

Prior to commencing fabrication, PTFE powder, PLA pellets, CNT, and Fe_3_O_4_ nanoparticles were dried in a vacuum oven at 85 °C for 10 h to remove moisture from the raw materials. For the preparation of the FCFe membranes with 50 wt% Fe_3_O_4_ content as an example, two portions of 9 g of PLA were first weighed and dissolved in 100 mL of DCM each. Then, 0.05 g of CNT and 1 g of Fe_3_O_4_ were separately added to the prepared PLA/DCM solution. After thorough stirring, the two solutions were combined in one beaker and subjected to ultrasonic treatment for 4 h to obtain the CNT/Fe_3_O_4_/PLA/DCM suspension with uniformly dispersed fillers. The mixture was then placed in a 70 °C water bath and heated with mechanical stirring for 4 h. Next, the mixture was transferred to a vacuum oven at 90 °C and dried for 24 h until the DCM was completely evaporated. After the solution was completely dried, the resulting solid material was cut into small pellets. Next, 1 g of PTFE was thoroughly mixed with the CNT/Fe_3_O_4_/PLA pellets, and the mixture was injected into a twin-screw extruder and processed at 190 °C for 10 min. The extruded PTFE/CNT/Fe_3_O_4_/PLA was then hot-pressed at 10 MPa and 190 °C to form a film. To ensure complete removal of PLA, the PTFE/CNT/Fe_3_O_4_/PLA film was subjected to Soxhlet extraction in DCM at 65 °C for 12 h, resulting in the formation of the FCFe membrane. By adjusting the mass fraction of Fe_3_O_4_, FCFe membranes with Fe_3_O_4_ contents of 10 and 30 wt% were also prepared.

### Synthesis of Ti_3_C_2_T_x_ MXene Dispersion

Ti_3_C_2_T_x_ MXene few-layer dispersion was prepared using a minimal delamination method [37]. First, 3.2 g of LiF were dissolved in 40 mL of 9 M HCl and stirred for 1 h. Then, Ti_3_AlC_2_ powder was slowly added to the mixture and stirred at 40 °C for 24 h. The resulting dispersion was repeatedly washed with deionized water until the pH of the supernatant was greater than 6. Such dispersion was sonicated for 40 min under nitrogen atmosphere. Finally, the mixture was centrifuged at 3500 rpm for 1 h, and the black supernatant obtained was the few-layer MXene dispersion.

### Preparation of the FCFe/M Janus Membranes

First, one side of the FCFe membrane is subjected to plasma treatment to enhance its hydrophilicity. Subsequently, a simple vacuum-assisted filtration process is employed to deposit dispersions of MXene of varying volumes onto the plasma-treated side. Finally, the prepared film is hot-pressed at 50 kPa for 30 min, resulting in the formation of the FCFe/M Janus membrane.

### Characterization and Measurements

The microstructure of the FCFe/M surfaces and cross sections was investigated using a field emission scanning electron microscope (FESEM, Gemini 500, Zeiss, Germany). The wide-angle X-ray diffraction (WAXD) of the samples was performed using an X-ray diffractometer (XRD, DMAX-2500PC, Rigaku, Japan) from 5° to 70° and a step interval of 5° per min. To analyze the chemical composition of the composite films, X-ray photoelectron spectroscopy (XPS, ESCALAB 250Xi, Thermo Scientific, USA) was employed. Prior to all tests, the samples were dried in an oven at 80 °C for 3 h. Following ASTM D792 standards, the sample densities were measured using an analytical balance (AB204, Mettler Toledo, Switzerland). The porosity (ϵ) of the FCFe membranes was quantified by measuring the absorption of n-butanol into the membrane pores, with porosity calculated using the following equation [38]:

1 $$\epsilon = \frac{{\left( {m_{1} - m_{2} } \right)/\rho_{1} }}{{\left( {m_{1} - m_{2} } \right)/\rho_{2} + m_{1} /\rho_{2} }}$$

where *m*_*1*_ represents the weight of the sample after being wetted with n-butanol, while *m*_*2*_ corresponds to the weight of the dried membrane. *ρ*_*1*_ denotes the density of n-butanol (0.8097 g mL^−1^), and *ρ*_*2*_ indicates the average density of all materials within the membrane. The membrane was cut into rectangular pieces measuring 50 mm × 10 mm and subjected to tensile testing on a universal testing machine (HDW-2000, Hengxu, China) at a speed of 1 mm min^−1^ to evaluate the mechanical properties of the FCFe/M. The magnetic hysteresis loop of the samples was measured using a vibrating sample magnetometer (7404, Lake Shore, American). The thermomechanical properties were characterized by a dynamic mechanical analyzer (Discovery DMA-850, TA, USA), where samples with dimensions of 25 mm × 5 mm were stretched over a temperature range of − 150 to 300 °C, with a heating rate of 5 °C min^−1^ and a frequency of 1 Hz. The hydrophilic/hydrophobic properties of the membrane were investigated using a contact angle measuring device (JC2000D, Powereach, China). Approximately 3 μL of deionized water droplets were deposited on the flat film surface, and their dynamic images were captured. The electrical conductivity of the samples was measured with a four-point probe tester (ST2242, Suzhou Jingge, China). The electromagnetic interference (EMI) shielding effectiveness (SE) of the membrane in the X-band frequency range was measured using a vector network analyzer (N5247A, Agilent, USA), with the sample placed in a rectangular waveguide (22.86 mm × 10.16 mm). Due to the softness of the FCFe/M membrane, it was secured in place using two 0.3-mm thick PC plates during the test. The EMI SE and power parameters of the samples were calculated based on *S*-parameters [16, 23]:

2 $$SE_{R} = - 10\log \left( {1 - \left| {S_{11}^{2} } \right|} \right)$$

3 $$SE_{A} = - 10\log \left( {\left| {S_{21}^{2} } \right|/\left( {1 - \left| {S_{11}^{2} } \right|} \right)} \right)$$

4 $$SE_{T} = {\text{SE}}_{{\text{R}}} + {\text{SE}}_{{\text{A}}} + {\text{SE}}_{{\text{M}}}$$

5 $$R = \left| {S_{11}^{2} } \right|$$

6 $$T = \left| {S_{21}^{2} } \right|$$

7 $$A = 1 - R - T$$

where *S*_*11*_ and *S*_*21*_ are scattering parameters. *SE*_*T*_ represents the total EMI SE, *SE*_*R*_ denotes the reflection SE, and *SE*_*A*_ indicates the SE due to absorption. The parameters *R*, *A*, and *T* refer to the power coefficients of reflection, absorption, and transmission of the sample, respectively. where the *SE*_*M*_ is negligible if the *SE*_*T*_ is greater than 10 dB. To compare the effectiveness of shielding materials equitably, specific shielding effectiveness (SSE) taking into account the density and thickness is represented as follows [5]:

8 $$SSE = \frac{{SE_{T} }}{\rho \times W}$$

where *ρ* represents the density of the membrane, and *W* denotes the thickness of the membrane. EMI shielding efficiency (%), referring to the capability to block waves in terms of percentage, is obtained using the following equation:

9 $${\text{Shielding}} {\text{efficiency}} \left( \% \right) = 100 - \left( {\frac{1}{{10^{{\frac{{SE_{T} }}{10}}} }}} \right) \times 100$$

The thermal diffusivity (*α*) was determined by HyperFlash (LFA 467, Netzsch, Germany) at 25 °C. The thermal conductivity (*λ*) was calculated by the following equation:

10 $$\lambda = \rho \times C_{p} \times \alpha$$

where *ρ* and *C*_*p*_ are the density and specific heat of the measured samples. The voltage for the electrical heating test was supplied by a digital source meter (2450, Keithley, USA). The UV–Vis-NIR absorption and reflection spectra of the FCFe/M membranes in the range of 200 to 2500 nm were measured using a spectrophotometer (Lambda 950, PerkinElmer, USA). Simulated sunlight was provided by a xenon lamp (CEL-HXF300-T3, Zhongjiao, China). All infrared thermal images were captured using an infrared camera (E8xt, FLIR, USA). The surface temperature of the samples was recorded using a digital thermometer (MIK-R200T, Supmea, China).

## Result and Discussion

### Fabrication and Characterization of the FCFe/M Janus Membranes

**Fig. 1:**
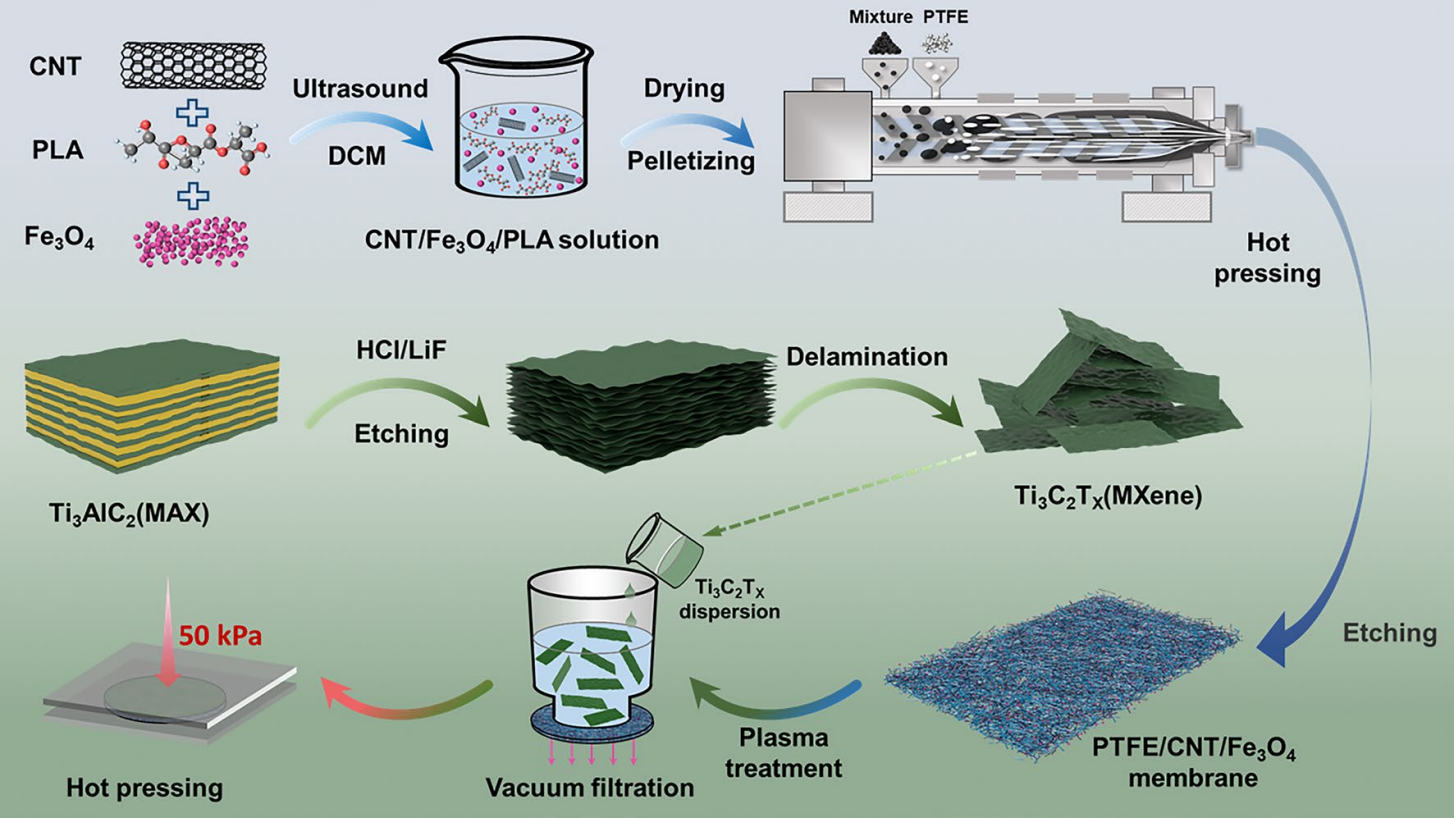
Schematic illustration of the preparation of FCFe/M Janus membranes based on an in situ fibrillation and vacuum-assisted filtration strategy

The preparation process of FCFe/M composite membranes is illustrated in Fig. 1. To ensure the uniform distribution of the modified fillers in the nanofibrous network, CNT and Fe_3_O_4_ were initially mixed with PLA in DCM. The mixture was then dried and pelletized into easily workable particles. The CNT/Fe_3_O_4_/PLA blends were subsequently thermally compounded with PTFE particles in a twin-screw extruder. During this process, the PLA melt acted as a lubricant, transferring the shear force generated by the screws to the PTFE particles. This shear mixing at a specific temperature avails the PTFE crystals to untangle and fibrillate into a nanofibrous structure [39, 40]. Meanwhile, CNT and Fe_3_O_4_ were uniformly dispersed into the PTFE/CNT/Fe_3_O_4_/PLA mixture. Following hot-pressed the mixture into a film, repeated etching with DCM was performed to completely remove the polylactic acid, yielding a purified FCFe membrane. As a result, PTFE fibers interweave with CNT fibers to form a dual-nanofibrous structure, tightly encapsulating the Fe_3_O_4_ nanoparticles. As shown in Fig. S1a, the XRD spectra of the resulting products distinctly display the characteristic peaks of Fe_3_O_4_ and CNT, confirming the successful preparation of FCFe composite membranes. To verify the uniform dispersion of CNT and Fe_3_O_4_ within the FCFe membranes, we measured conductivity and saturation magnetization values at 6 different orientations and positions of the membrane (Table S1). The results showed that the conductivity variation did not exceed 6%, and the saturation magnetization variation was less than 5%. These findings confirm the homogeneous distribution of CNT nanofibers and Fe_3_O_4_ nanoparticles within the PTFE nanofibrous network.

Ti_3_AlC_2_ was utilized to synthesize Ti_3_C_2_T_x_ MXene nanosheets, where the Al layer in Ti_3_AlC_2_ was selectively etched using an HCl and LiF solution mixture, followed by sonication and centrifugation of the resulting precipitate. The characteristic peak of Ti_3_AlC_2_ layer spacing appeared at 9.5° (002), and the characteristic peak of Al layer appeared at 38.9°. After acid etching, the (002) peak shifted to 6.7°, while the other characteristic peaks nearly vanished, confirming the successful preparation of MXene (Fig. S1a) [37]. Subsequently, the prepared MXene dispersion was subsequently deposited on one side of the plasma-treated FCFe membrane by vacuum-assisted filtration. Finally, the FCFe/M Janus membrane underwent thermal pressing to further enhance its structural integrity and mechanical strength. Here, the FCFe/M membranes with the addition of 10, 30, 50, and 70 wt% of MXene relative to the total mass of the FCFe membrane are defined as FCFe/M-10 m, FCFe/M-30 m, FCFe/M-50 m, and FCFe/M-70 m, respectively.

**Fig. 2:**
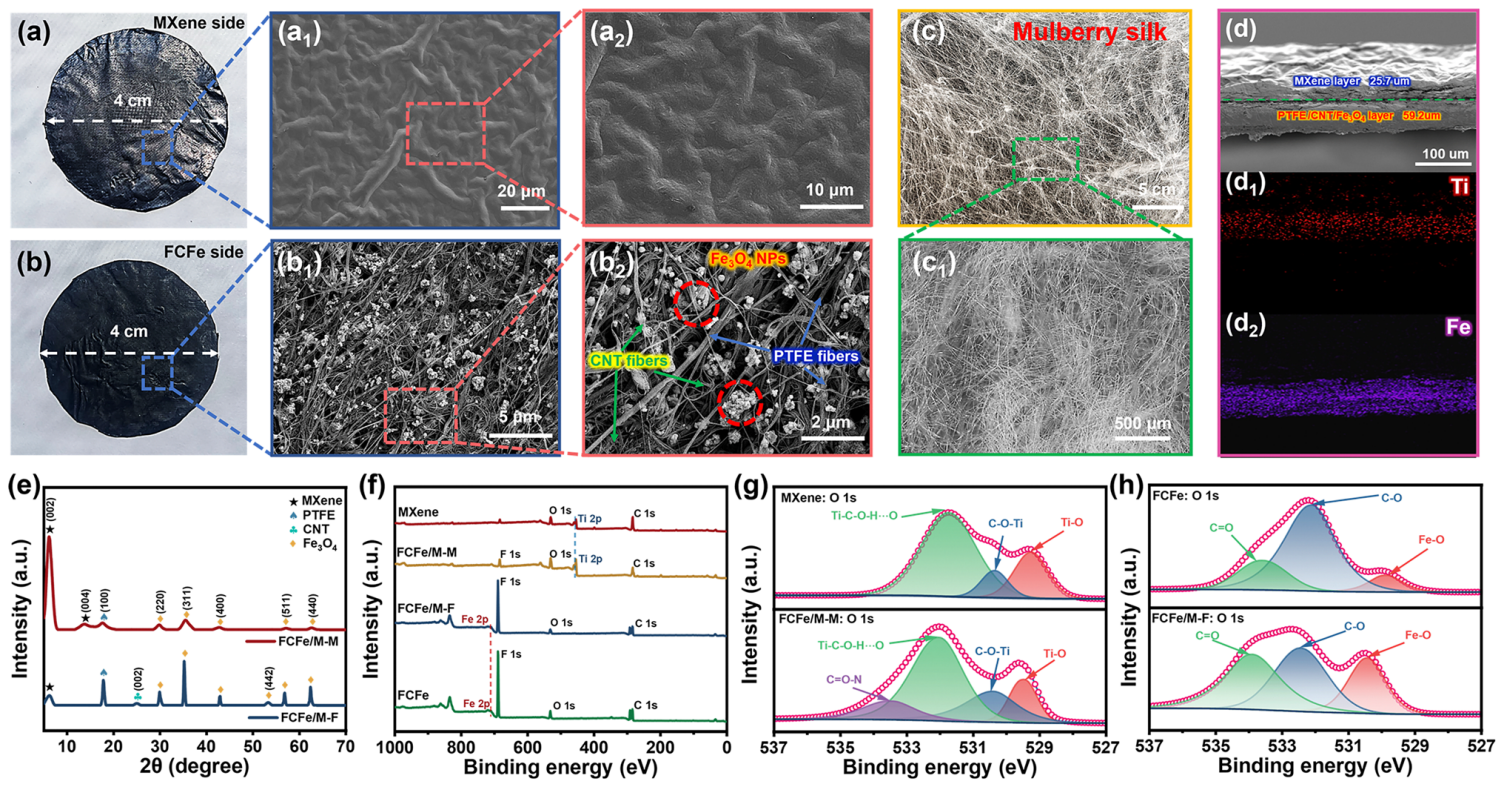
Characterization and microstructures of FCFe/M membranes. Digital photos of **a** FCFe/M-M and **b** FCFe/M-F. Surface morphology of **a**_**1**_**, a**_**2**_ FCFe/M-M and **b**_**1**_**, b**_**2**_ FCFe/M-F. The **c** photograph and **c**_**1**_ microscopic image of mulberry silk. **d** SEM image and **d**_**1**_**, d**_**2**_ elemental mapping of the cross section of FCFe/M Janus membrane. **e** XRD patterns of FCFe/M-M and FCFe/M-F. **f** XPS spectra of MXene, FCFe/M-M, FCFe/M-F, and FCFe. **g** O 1s spectra of MXene and FCFe/M-M. **h** O 1*s* spectra of FCFe and FCFe/M-F

Figure 2a, b illustrates the macroscopic optical images and microscopic structure of FCFe/MXene membranes. Interestingly, although the FCFe/M membrane has an overall black appearance, its two surfaces exhibit distinct visual properties discernible to the naked eye. The MXene side displays a wrinkle-like structure with a metallic luster, attributed to the stacking of layered nanosheets (Fig. 2a_1_, a_2_). Conversely, the FCFe side has a rough, lusterless texture. As shown in Fig. 2b_1_, b_2_, the coarser nanofibers are identified as PTFE, while the thinner, curly nanofibers are CNT. These CNT nanofibers are randomly and uniformly distributed within the gaps of the PTFE nanofibers. Figure S1b illustrates that the diameter of PTFE nanofibers is mostly distributed in the range of 100–200 nm, whereas the CNT nanofibers exhibit a finer and more concentrated diameter of below 50 nm. These two types of nanofibers intertwine to construct a porous and robust dual-nanofibrous structure, leveraging the advantages of both components. Based on these structural characteristics, the lustrous MXene side is designated as FCFe/M-MXene (FCFe/M-M), while the rough FCFe side is designated as FCFe/M-FCFe (FCFe/M-F). Further examination of the internal microstructure of FCFe/M-F reveals that the fully fibrillated PTFE fibers interweave with finer CNT fibers, forming a silk-like nanofibrous network (Fig. 2c, c_1_). The silk-like structure not only securely traps Fe_3_O_4_ nanoparticles, but also effectively prevents the infiltration of MXene nanosheets into the FCFe layer, ensuring structural integrity and functional segregation. Notably, owing to the porous structure formed by the silk-like nanofibrous network, the FCFe membrane achieves a porosity of up to 71.2% (Fig. S1c). With increasing Fe_3_O_4_ content, the porosity of the composite membranes exhibited a gradual increase. It is speculated that the Fe_3_O_4_ nanoparticles dispersed within the nanofibrous structure gradually props up the silk-like network, leading to the formation of smaller and more numerous pores (Fig. S2) [35]. Figures 2d and S3 show the cross-sectional microstructure of the FCFe/M Janus membrane. The composite membrane displays a distinctly differentiated layered structure, where the dense MXene layer (25.7 µm) is tightly bonded to the FCFe supporting layer (59.2 µm) through hydrogen bonding and van der Waals forces. Furthermore, energy-dispersive X-ray spectroscopy (EDS) analysis of the FCFe/M membrane shows that Ti elements are primarily concentrated in the MXene layer, while Fe elements are predominantly located in the FCFe layer, further confirming the successful fabrication of the Janus structure (Fig. 2d_1_, d_2_). The advantage of this Janus configuration lies in the FCFe layer acting as a substrate, providing the composite membranes with enhanced membrane-forming ability and mechanical properties, while the MXene layer enriches the opposite side, delivering excellent electrical conductivity.

To better understand the formation mechanism of the FCFe/M, additional tests were conducted to analyze the chemical structures and validate the role of hydrogen bonding interactions between the MXene layer and the FCFe layer. As shown in Fig. 2e, the XRD patterns of both sides of the FCFe/M Janus membrane display characteristic peaks corresponding to MXene, PTFE, CNT, and Fe_3_O_4_. Notably, compared to pure MXene, the peak representing the (200) crystal plane in the FCFe/M-M layer shifts to a lower angle (from 6.7° to 6.2°), indicating that the FCFe layer is tightly bonded to the MXene layer through strong interactions [41]. Figure 2f presents the XPS wide-scan spectra of MXene, FCFe, and FCFe/M membranes. The MXene spectrum shows the presence of C, O, Ti, and F elements, with no detectable Al, indicating that the Al layer in Ti_3_AlC_2_ has been fully etched away with the introduction of functional groups (− O, − OH, − F) [42]. Notably, the XPS spectrum of FCFe/M-F closely resembles that of FCFe, while FCFe/M-M mirrors the MXene spectrum, further confirming the intrinsic Janus characteristics of the FCFe/M membrane [43]. Upon meticulous peak fitting analysis, it is found that in the O 1*s* spectrum of MXene, the peaks corresponding to Ti − O, C − O − Ti, and Ti − C − O − H…O were observed at 529.22, 530.27, and 531.81 eV, respectively. In contrast, in the O 1*s* spectrum of FCFe/M-M, these peaks shift slightly to 529.40, 530.43, and 532.03 eV, respectively, and a new feature peak appears at 533.48 eV, representing C = O − N (Fig. 2g). This newly observed peak may be attributed to the interaction between MXene and FCFe, which provides strong support for the stability of the Janus structure [44, 45]. Interestingly, similar peak shifting phenomena are also observed on the other side of the Janus membrane (Figs. 2h and S4). Compared to FCFe, the binding energy of the Fe − O, C − O, and C = O peaks in FCFe/M-F shift to higher values by 0.27, 0.32, and 0.47 eV, respectively. The slight upshift of O 1*s* binding energy of FCFe/M-M further confirms the formation of hydrogen bonds between MXene and FCFe [37, 44, 46]. This differential coordination interaction, originating from the hydrogen bonds and van der Waals forces between the FCFe layer and MXene layer, imparts a stable Janus structure to the composite membrane and enhances the mechanical properties of FCFe/M to some extent [47].

### Mechanical Properties, Thermal Stability, Hydrophobicity and Flame Retardancy of the FCFe/M Janus Membranes

**Fig. 3:**
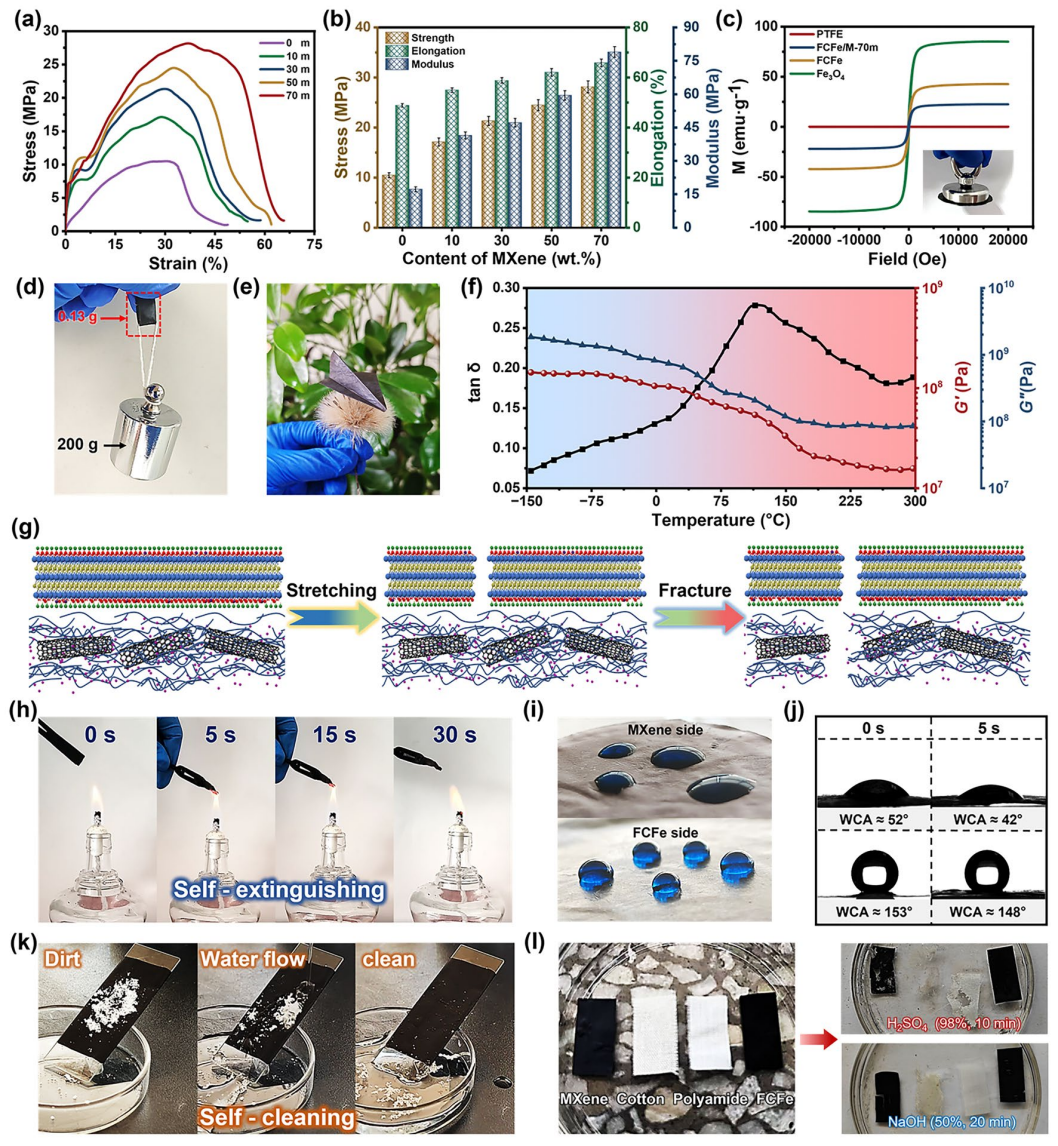
Characterization and macroscopic properties of FCFe/M membranes. **a** Stress–strain curves of FCFe/M membranes with different MXene contents. **b** Statistics of tensile stress, elongation at break, and Young’s modulus the FCFe/M membranes with different MXene contents. **c** Hysteresis loops of PTFE, Fe_3_O_4_, FCFe, FCFe/M-70 m. (The illustration shows a magnet attracting the FCFe/M membrane.) **d** Photograph of a 0.13 g FCFe/M-70 m membrane withstanding a 200 g weight. **e** Photograph shows an origami plane made from a FCFe/M membrane rest on a dandelion. **f** DMA curves of the FCFe/M-70 m membrane. **g** Schematic diagram of fracture mechanism of the composite membranes. **h** Digital photograph of the FCFe/M-70 m membrane under an alcohol lamp. **i** Digital photographs of water droplet and **j** water contact angles on the surface of MXene side and FCFe side. **k** Self-cleaning function shown by cleaning the dirt upon the textile. **l** Comparison of corrosion resistance of MXene, FCFe and commercial textiles

Superior mechanical performance is a prerequisite for the application of FCFe/M membranes in flexible wearable technologies. Leveraging the inherent mechanical strength of PTFE fibers and the robust interfacial interactions between the FCFe and MXene layers, FCFe/M Janus membranes demonstrate exceptional mechanical performance and outstanding flexibility. As depicted in Fig. S5 and Table S2, compared to pure PTFE film, the tensile strength of FCFe membranes is significantly enhanced, which can be attributed to the formation of the dual-nanofibrous network composed of CNT fibers and PTFE fibers. With the increase in Fe_3_O_4_ content, the mechanical performance of FCFe membranes declines, which can be attributed to the disruption of molten interfaces between PTFE fibrils and the increased membrane porosity [35]. Furthermore, the incorporation of MXene into the composite membrane resulted in a marked improvement in tensile strength, elongation at break, and elastic modulus compared to the original FCFe membrane (Fig. 3a, b). Of particular interest is the emergence of an elastic-like deformation region with rapidly increasing stress during the initial deformation phase of the FCFe/M Janus membrane, a characteristic absent in the pure FCFe membrane. This phenomenon is likely attributed to the initial stress being effectively absorbed by the FCFe/M-M layer, highlighting the enhanced mechanical properties imparted by the MXene integration [26]. As the MXene content in the FCFe/M composite membranes increased from 10 to 70 wt%, the tensile strength of the composite membrane gradually increased from 17.11 to 28.14 MPa. Similarly, the elongation at break exhibited a slow yet steady rise, from 54.89% to 65.89%. Additionally, the modulus improved significantly, increasing from 41.47 to 78.95 MPa. The detailed mechanical properties are summarized in Table S2. These results indicate that MXene not only enhances its own mechanical strength through its compactly stacked layered structure, but also forms effective interlocking with the nanofibrous network of the FCFe membrane via hydrogen bonding [37, 45]. These two factors work synergistically to further reinforce the Janus structure beyond the elastic deformation phase, thereby enhancing the overall mechanical performance of the composite.

Figure 3c shows the magnetic hysteresis loops of the films. Pure PTFE exhibits no magnetic properties, whereas both FCFe and FCFe/M-70 m membranes demonstrate typical ferrite characteristics similar to Fe_3_O_4_, with saturation magnetization values of 42.45 and 22.24 emu g^−1^, respectively. This exceptional magnetic performance allows the FCFe/M-70 m membrane to be easily lifted by a magnet. Figure 3d demonstrates that the lightweight FCFe/M-70 m membrane, with a mass of only 0.13 g, can effortlessly support a 200 g load without any signs of cracking or failure, highlighting its impressive mechanical properties. Moreover, the membrane’s remarkable flexibility enables it to be bent or folded into complex shapes (Fig. 3e). To assess performance under extreme conditions, the thermomechanical properties of the FCFe/M-70 m membrane were further evaluated. As shown in Fig. 3f, the FCFe/M membrane demonstrates exceptional durability and stability across a wide temperature range, from − 150 to 300 °C. This remarkable resistance to heat and cold can be attributed to its regular molecular structure, stable C–F bonds with high bond energy, and relatively high molecular weight [35].

To further elucidate the reinforced toughening mechanism of FCFe/M Janus-structured membranes, a possible fracture process is intuitively proposed. As shown in Fig. 3g, under low tensile strain, the strong interfacial interactions formed by hydrogen bonds between the MXene layer and the FCFe layer enhance stress transfer and frictional energy dissipation, effectively inhibiting slippage and crack propagation of MXene nanosheets [48]. During further stretching, the MXene layer in the FCFe/M Janus membrane is the first to develop cracks and undergo failure. However, the polymer chains in the FCFe layer undergo slippage under stress, which effectively impedes further crack propagation and preserves the overall integrity of the FCFe/M membrane [39]. Ultimately, as the stress and strain continue to accumulate and surpass a critical threshold, the silk-like structure within the FCFe layer begins to progressively break down.

Many conventional CPCs exhibit high flammability, making them prone to fire hazards in EMI shielding applications, which poses significant risks to equipment and even personnel [49]. In this regard, the FCFe/M membranes exhibit self-extinguishing and melt-drip resistance properties. As demonstrated in Fig. 2h and Video S1, the FCFe/M-70 m membrane produced only minimal residue after 30 s of combustion and extinguished immediately upon removal from the flame source within 5 s. The excellent flame retardancy of the FCFe/M membranes can be attributed to two factors. Firstly, the intrinsic flame-retardant properties of PTFE and CNT contribute significantly [35]. Secondly, the TiO_2_ and amorphous carbon generated from the oxidative decomposition of MXene during combustion form a protective shielding layer that efficiently mitigates both flame propagation and energy transfer [50]. Additionally, excellent hydrophobicity and corrosion resistance are also crucial for ensuring the long-term stability of flexible wearable electronics under harsh environmental conditions. MXene materials, due to their inherent hydrophilicity, are highly susceptible to oxidation in humid environments, which significantly degrades their performance [51]. As depicted in Fig. 3i, j, the abundant functional groups (–F, –OH) on the MXene surface impart notable hydrophilicity to the FCFe/M-M, resulting in a water contact angle (WCA) of only 52°. In contrast, the FCFe/M-F surface, benefiting from the exceptionally low surface tension of PTFE and the rough surface texture provided by Fe_3_O_4_ nanoparticles, achieves a water contact angle of 153°, demonstrating superhydrophobicity. After standing for 5 s, the WCA on the FCFe side decreased by 5°, which is only half of the reduction observed on the MXene side. This remarkable hydrophobicity can be attributed to the low surface energy of PTFE [39]. Consequently, the FCFe/M membranes demonstrate exceptional water resistance, with water droplets effortlessly rolling off the FCFe side and carrying away contaminants without leaving any residue, showcasing superior rain resistance and self-cleaning capabilities (Fig. 3k). Additionally, due to the excellent solvent resistance of PTFE and CNT, the FCFe membranes exhibit strong durability under various harsh chemical conditions. As shown in Fig. 3l, under acidic and alkaline conditions, the integrity of the pure MXene membrane was compromised, and traditional textiles such as cotton and polyester also underwent substantial decomposition. In stark contrast, the FCFe membrane remained intact without any signs of destruction. Therefore, during the operation of the FCFe/M Janus membrane, simply exposing the FCFe side to the external environment can effectively protect the MXene conductive layer, thereby extending the lifespan of electronic devices.

### Electrical Properties and EMI Shielding Performance of the FCFe/M Membranes

**Fig. 4:**
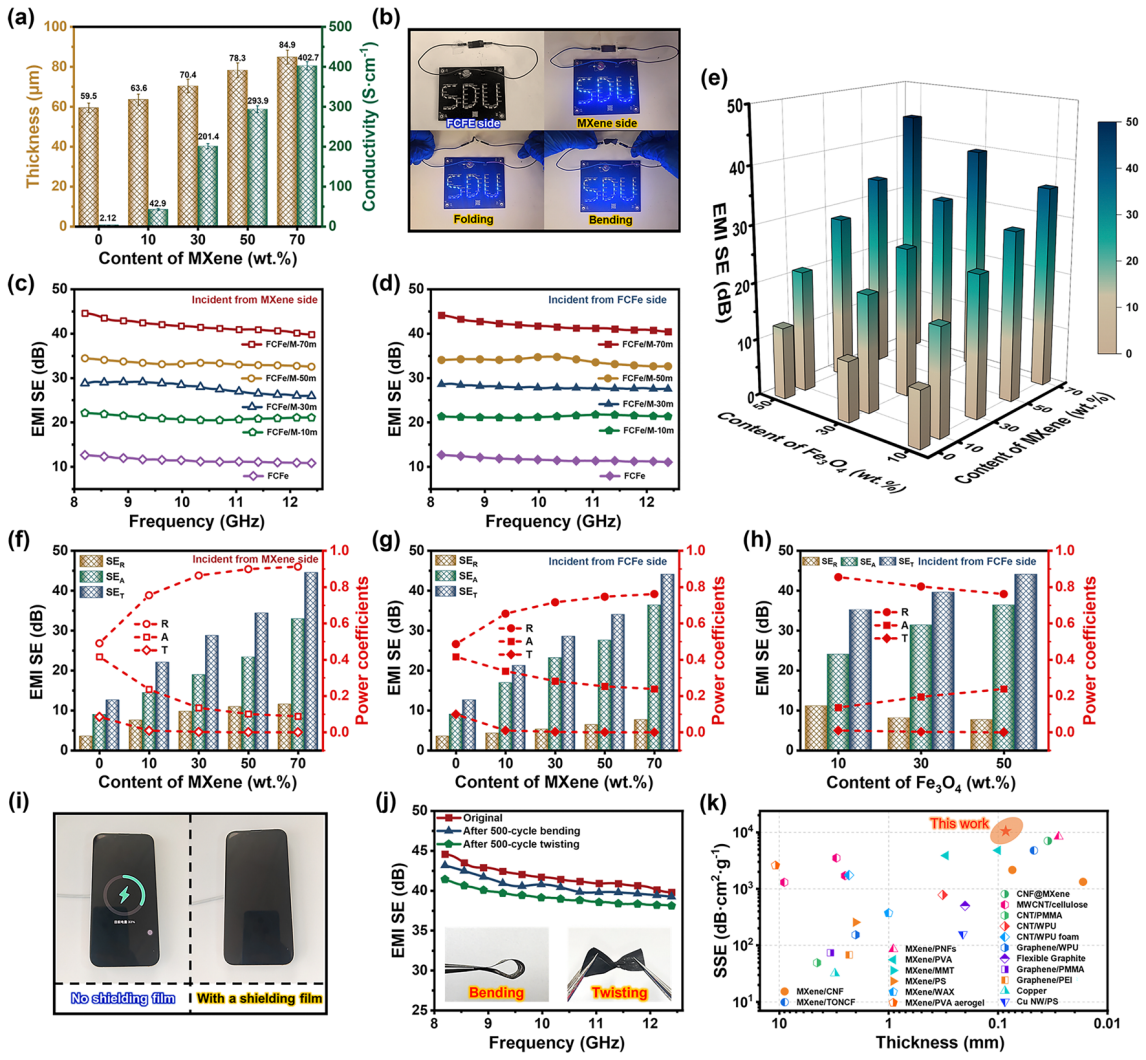
Electrical properties and EMI shielding performance of FCFe/M membranes. **a** The thickness and electrical conductivity of FCFe/M Janus membranes with different MXene contents. **b** The digital images show the variation in brightness of a small light bulb under different test conditions. The EMI SE of FCFe/M membranes in the X-band when incident from **c** the MXene side and **d** the FCFe side. **e** 3D bar chart of the EMI SE for FCFe/M membranes with varying MXene and Fe_3_O_4_ content. EMI shielding performance (*SE*_*T*_, *SE*_*A*_, and *SE*_*R*_) and power coefficients (*R*, *T*, and *A*) of FCFe/M membranes when incident from **f** the MXene side and **g** FCFe side. **h** EMI shielding performance and power coefficients of FCFe/M membranes with different Fe_3_O_4_ contents. **i** Photographs demonstrate that the FCFe/M-70 m membrane effectively blocks the transmission of radio waves. **j** EMI SE of the FCFe/M-70 m membrane before and after bending and twisting. **k** Comparison of EMI shielding performance (SSE) and thickness of the FCFe/M-70 m membrane with typical shields reported

Theoretically, the performance of EMI shielding materials is tightly correlated with its electrical conductivity and internal microstructure. Thanks to the formation of well-connected MXene conductive networks, FCFe/M membranes exhibit distinct electrical properties on two sides. Figure 4a illustrates the conductivity of FCFe/M membranes. Without the addition of MXene, the electrical conductivity of the pure FCFe membrane is only 1.12 S cm^−1^. However, as the MXene content increases from 10 to 70 wt%, the conductivity undergoes a dramatic surge, rising from 42.71 S cm^−1^ to an impressive 402.78 S cm^−1^. The significant disparity in conductivity between the two sides of the Janus FCFe/M-70 m membrane enables it to control the light bulb’s switching (Fig. 4b). Additionally, the exceptional flexibility of composite membranes, combined with robust interfacial interactions, significantly strengthens the conductive network within the MXene layer, allowing the LED light to maintain stable brightness even when the film is repeatedly bent or folded.

Benefiting from the distinctive Janus structure created by the conductive reflective layer (MXene) and the magnetic absorption layer (FCFe), linked via hydrogen bonding and electrostatic interactions, FCFe/M membranes effectively attenuates EMWs through the synergistic effects of conductive and magnetic losses. Figure 4c, d illustrates the EMI shielding performance of FCFe/M membranes with varying MXene content in the X-band. The pure FCFe membrane demonstrates a relatively low EMI SE of only 12.64 dB, reflecting suboptimal shielding capability. However, as the MXene content increases, the shielding performance of the FCFe/M Janus membranes progressively improves, achieving a maximum EMI SE of 44.56 dB at 70 wt% MXene, significantly surpassing the commercial requirements (> 20 dB). The increase in MXene content significantly enhances the ability of FCFe/M membranes to shield EMWs. Furthermore, to explore the impact of magnetic particles on the overall shielding performance, the EMI SE of FCFe/M membranes with varying Fe_3_O_4_ content was tested (Fig. 4e). As anticipated, the EMI SE of FCFe/M membranes increases with the rising Fe_3_O_4_ content, confirming the critical role of magnetic loss in the EMI shielding process (Fig. S6).

Due to the different electrical conductivities on either side of FCFe/M Janus membranes, an investigation was conducted to ascertain the impact of EMW incidence direction on the membrane’s EMI shielding performance. Obviously, the EMI SE of the FCFe/M membranes remains consistent, regardless of whether the EMWs are incident from the MXene side or the FCFe side. It suggests that the direction of incidence of the EMWs has no impact on the total shielding efficiency (*SE*_*T*_) of the FCFe/M Janus membranes, without considering the EMI shielding mode. However, the perpendicular incidence direction of EMWs can affect the reflective shielding efficiency (*SE*_*R*_) and absorption shielding efficiency (*SE*_*A*_). As illustrated in Fig. 4f, g, the *SE*_A_ of all the samples exceeds their respective *SE*_*R*_, suggesting that microwave absorption has a significant predominant contribution to the *SE*_*T*_ [52]. That is to say, without considering the initial reflection, most of the EMWs entering the FCFe/M membranes are absorbed internally [53]. As the MXene loading increases, although the *SE*_*A*_ shows a general upward trend for both incident directions, the rate of increase is significantly higher when the waves are incident from the FCFe side compared to when they are incident from the MXene side. Additionally, the proportion of *SE*_*A*_ in *SE*_*T*_ is greater when waves are incident from the FCFe side. It is hypothesized that when EMWs enter the FCFe/M membrane from the FCFe side, a substantial portion of the waves is absorbed within the FCFe layer through conductive and magnetic losses. In contrast, when EMWs are incident from the MXene side, they encounter a severe impedance mismatch at the MXene layer, leading to significant reflection back into the air, with only a small fraction penetrating into the FCFe layer.

Nevertheless, previous studies have demonstrated that relying solely on *SE*_*R*_ and *SE*_*A*_ to classify shielding modes is insufficient and inaccurate [16]. To provide a more comprehensive understanding of the shielding mechanisms in FCFe/M membranes and to assess the differences in antireflective properties under various incident directions, the reflection coefficients (*R*), absorption coefficients (*A*), and transmission coefficients (*T*) were measured (Fig. 4f, g). The result shows that in the absence of MXene, the *T* value is relatively high, indicating that the FCFe membrane exhibits suboptimal shielding performance. As the MXene content increases, the *R* values show a marked increase, while the *A* values tend to decrease, and the *T* values progressively decline, stabilizing near zero. This trend highlights that an increase in MXene content significantly enhances the reflective capability of the membranes, thereby optimizing its overall shielding performance. When EMWs are incident from the MXene side, the *R* value for FCFe/M-70 m is 0.91, which exceeds the value of 0.76 observed when waves are incident from the FCFe side. Conversely, the *A* value is 0.09 for waves incident from the MXene side, markedly lower than the 0.24 recorded for waves incident from the FCFe side. These discrepancies in loss factors between different incident directions are primarily due to the varying impedance matching between the two sides of the FCFe/M Janus membrane and the free space. Specifically, when waves enter the FCFe/M membrane from the lower conductivity FCFe side, a significant portion is absorbed within the FCFe layer. The remaining waves are then reflected at the MXene/FCFe interface and reenter the FCFe layer, effectively prolonging their path within the absorbing layer and thereby enhancing the film’s antireflective performance. In FCFe/M-70 m membrane, the *R* value is higher than the *A* value, indicating that the reflection is the dominant EMI shielding mechanism. The high-power coefficient *R* should be due to the fact that the reflection of incoming EMWs happens before the absorption, which results in most of the total power is reflected first when it comes into contact with the membrane surface (Although the *SE*_*R*_ of FCFe/M membrane is much lower than *SE*_*A*_).

Further analysis of the effect of magnetic Fe_3_O_4_ nanoparticles on the EMI shielding performance of FCFe/M membranes shows that, as the Fe_3_O_4_ content in the FCFe layer increases from 10 to 50 wt%, the EMI SE of FCFe/M increases by 8.86 dB, with *SE*_*A*_ and *A* values rising by 51.2% and 75.1%, respectively (Fig. 4h). This trend indicates that the magnetic loss generated by Fe_3_O_4_ nanoparticles significantly enhances the EMW absorption capacity of the FCFe layer. The response of the FCFe layer to EM waves also can be expressed in terms of complex permittivity (ε), complex permeability (μ), and tangential loss (tan δ_ɛ_ = ε″/ε′ and tan δ_μ_ = μ″/μ′). In general, the real part (ε′) and imaginary part (ε″) of the complex permittivity correspond to the storage and consumption of electrical energy, and the real part (μ′) and imaginary part (μ″) of the complex permeability indicate the storage and dissipation of the magnetic energy [54]. As shown in Fig. S8a, b, the fluctuation ranges of ε' and ε″ are 8.52–11.77 and 7.46–8.53, respectively, and μ′ and μ'' are 0.47–1.04 and 0.028–0.12, respectively (Fig. S7a, b). A higher frequency will intensify the hysteresis of dielectric polarization, which is completely consistent with the trend of ε′ and ε″ decreasing with the increase in frequency. The relationship of tan *δ*_ɛ_ greater than tan *δ*_μ_ indicates that dielectric loss is dominant in EM loss, while magnetic loss plays an important role in enhancing loss (Fig. S7c).

Notably, the EMI SE results indicate that the Janus membrane is capable of shielding up to 99.997% of EMWs. Consequently, the FCFe/M-70 m membrane, with a thickness of only 84.9 µm, can effectively impede the transmission of radio energy and halt the charging process (Fig. 4i). Moreover, due to the inherent flexibility of the FCFe substrate, the FCFe/M-70 m membrane retains 96.83% and 92.57% of its original *SE*_*T*_ even after 500 cycles of bending and folding, respectively, demonstrating its remarkable stability in EMI shielding performance under complex mechanical environments (Fig. 4j). In consideration of lightweight, flexible nature, and thin profile of the FCFe/M membrane, the SSE values were further calculated with the goal of achieving efficient EMI shielding using minimal material. The results revealed that with 70 wt% MXene content, the SSE value of the FCFe/M membrane reached an impressive 10,421.3 dB cm^2^ g^−1^, surpassing many typical EMI shielding materials ever reported, including CNT-based, graphene-based, MXene-based, and metal-based films (Fig. 4k and Table S3).

**Fig. 5:**
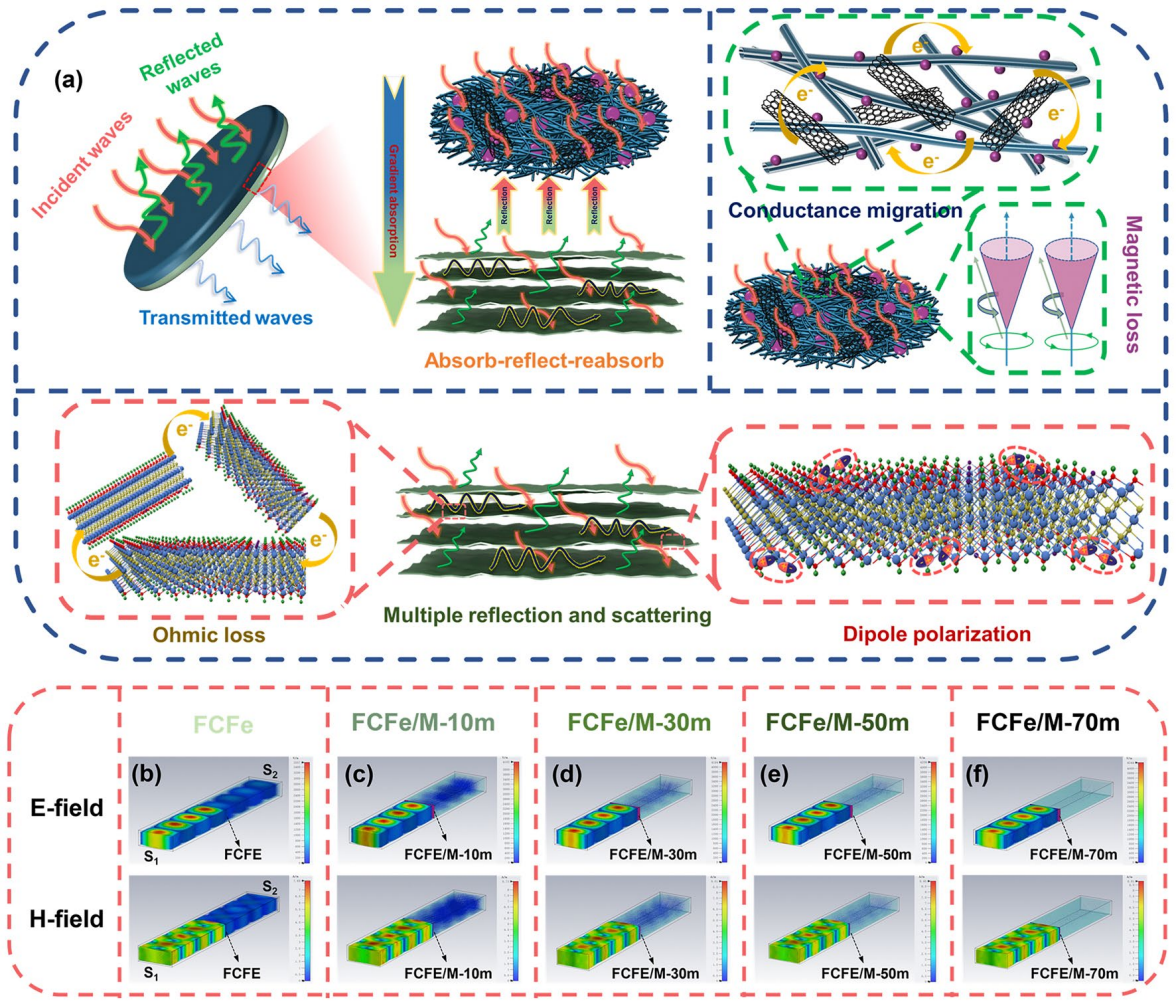
EMI shielding mechanism and visual simulation of the shielding performance of FCFe/M membranes. **a** EMI shielding mechanism of FCFe/M membranes, including impedance mismatch, magnetic loss, multiple reflections and scattering, ohmic loss, and polarization loss. CST simulation of **b** FCFe, **c** FCFe/M-10 m, **d** FCFe/M-30 m, **e** FCFe/M-FCFe/M-50 m, and **f** FCFe/M-70 m in X-band

In order to elucidate the EMI shielding mechanism of FCFe/M membranes, the attenuation process of EMWs is plotted in Fig. 5a. When EMWs are incident from the MXene side, the shielding performance is predominantly governed by the strong reflection induced by the highly conductive MXene layer, resulting in a higher *R* value and a lower *A* value. Conversely, when EMWs are incident from the FCFe side, the lower conductivity of the FCFe layer permits waves to penetrate more readily into the absorption layer. Subsequently, thanks to the silk-like nanofibrous structure composed of CNT, Fe_3_O_4_, and PTFE, a portion of the incident EMWs is dissipated as thermal energy while moving along the conductive fiber network. Additionally, multiple reflections and scattering within the silk-like nanofibrous network significantly extend the transmission path of these waves, effectively attenuating EM energy. Combined with the magnetic loss effect introduced by Fe_3_O_4_ nanoparticles, various shielding mechanisms collectively contribute to the excellent EMI absorption performance of the FCFe membrane [55]. When the EMWs penetrate the FCFe layer and reach the MXene layer, a portion of the remaining waves is immediately reflected back into the FCFe layer due to the impedance mismatch at the interface. These reflected waves are then reabsorbed within the FCFe layer [56], leading to the formation of an absorption-reflection-reabsorption mechanism [57]. Simultaneously, a smaller fraction of the waves manages to enter the MXene layer, where they interact with the high-density electron carriers present in the material. During this process, electron collisions generate thermal energy, leading to ohmic losses. Additionally, multiple reflection’s internal between adjacent MXene nanosheets also promoted the dissipation and attenuation of EMWs [58]. Moreover, defects and oxygen-containing functional groups in MXene can induce an asymmetric distribution of charge density, creating local dipoles. These dipoles undergo polarization in response to the direction of the EM field, resulting in an improvement in shielding effectiveness [37]. Therefore, by strategically controlling the direction of incidence, the FCFe/M Janus membrane can efficiently absorb EMWs through an absorption-reflection-reabsorption mechanism, even with a limited material thickness. This design, asymmetric gradient multilayer structure, provides an effective strategy for development of materials with ultrahigh EMI shielding performance.

CST simulations were conducted to elucidate and visualize the EMI shielding mechanism, with corresponding parameters detailed in Supporting S1 and Fig. S8. As depicted in Fig. 5b, for the FCFe membrane lacking a conductive reflective layer, EMWs readily penetrate the material from the excitation source S_1_ to the receiving source S_2_, indicating its poor EMI shielding capability. However, after depositing a high-conductivity MXene layer onto the FCFe substrate, there is a significant reduction in both electric and magnetic field strengths between the FCFe/M membrane and the receiving source S_2_ (Fig. 5c-e). When the MXene content is further increased to 70 wt%, the electric and magnetic fields nearly disappear, demonstrating the strong interaction between the EMWs and the FCFe/M-70 m membrane (Fig. 5f). Notably, all FCFe/M membranes respond to both electric and magnetic fields, indicating that the attenuation mechanism of EMWs involves not only conductive losses (including reflection and absorption), but also magnetic losses [5, 59]. Furthermore, the theoretical *SE*_*T*_ value of the FCFe/M-70 m membrane was calculated based on its thickness and conductivity (S2) [60, 61]. As shown in Fig. S9, the theoretical *SE*_*T*_ of the FCFe/M-70 m membrane with identical thickness and electrical conductivity is only ≈38.61 dB, which is significantly lower than the experimentally measured value. The additional EMI shielding contribution may stem from insulating structures within the FCFe membrane, beyond the conductive network. Typically, conductive fillers in the conductive polymer interconnect to form a percolation network, while some isolated fillers act as polar plates, together with the intermediate polymer matrix as the dielectric layer, constitute pseudo–parallel plate microcapacitors in composites. Since the CNT content in the FCFe membrane is only 5 wt%, the membrane lacks a complete percolation network. As a result, more insulating microcapacitors can form within the structure, which are coupled by EM waves to yield a shielding effect [62].

### Thermal Conductivity and Joule Heating Properties of FCFe/M Membranes

To further expand the application of EMI shielding materials in the field of flexible wearable technology, and to ensure their efficient performance in cold environments such as aerospace and plateaus, an excellent integrated heating capability is a requisite component [63]. Therefore, we first evaluated the thermal conductivity and thermal diffusivity of PTFE, FCFe, and FCFe/M membranes. As shown in Fig. S10a, compared to the pure PTFE film, the introduction of CNT, Fe_3_O_4_, and MXene significantly enhanced the thermal performance of the FCFe/M membrane. The in-plane thermal conductivity and diffusivity of FCFe/M-70 membranes reached 19.89 W m^−1^ K^−1^ and 9.11 mm^2^ s^−1^, respectively, which are 86.5 and 65.1 times higher than those of pure PTFE membranes. This improvement is primarily attributed to the silk-like nanofibrous network formed by CNT fibers, PTFE fibers, and Fe_3_O_4_ in the FCFe membrane, as well as the introduction of the 2D thermally conductive MXene material. These two components establish effective thermal conduction paths on both sides of the FCFe/M Janus membrane, which effectively reduces phonon scattering, strengthens phonon propagation, thereby enabling rapid heat diffusion throughout the membrane in the in-plane direction [26]. Figure S10b shows the through-plane thermal conductivity performance of the FCFe/M membrane. Similarly, with the introduction of modified fillers and 2D MXene materials, the through-plane thermal conductivity and thermal diffusivity of the membrane gradually increase, reaching a maximum of 1.92 W m^−1^ K^−1^ and 0.88 mm^2^ s^−1^, respectively. However, compared to in-plane thermal conductivity, the through-plane thermal conductivity of the FCFe/M membrane is significantly lower, with an anisotropic thermal conductivity value of 10.35. When heat flows along the through-plane direction, phonons scatter at the interfaces of the Janus structure, resulting in a significant loss of heat during the transmission process [26]. To evaluate the thermal performance of FCFe/M membranes in practical applications, we heated PTFE, FCFe, and FCFe/M membranes on an 80 °C hot plate for 30, 60, and 90 s, followed by rapid cooling to room temperature (Fig. S10c, d). The results demonstrate that the FCFe/M membrane exhibits the fastest heating and cooling rates, with a uniform temperature distribution throughout the membrane. In summary, the FCFe/M Janus membrane, which features high in-plane thermal conductivity and low through-plane thermal conductivity, is capable of quickly dissipating heat from hotspots in the plane direction, while preventing overheating from affecting the electronic components or skin beneath the FCFe/M Janus membrane [64, 65].

**Fig. 6:**
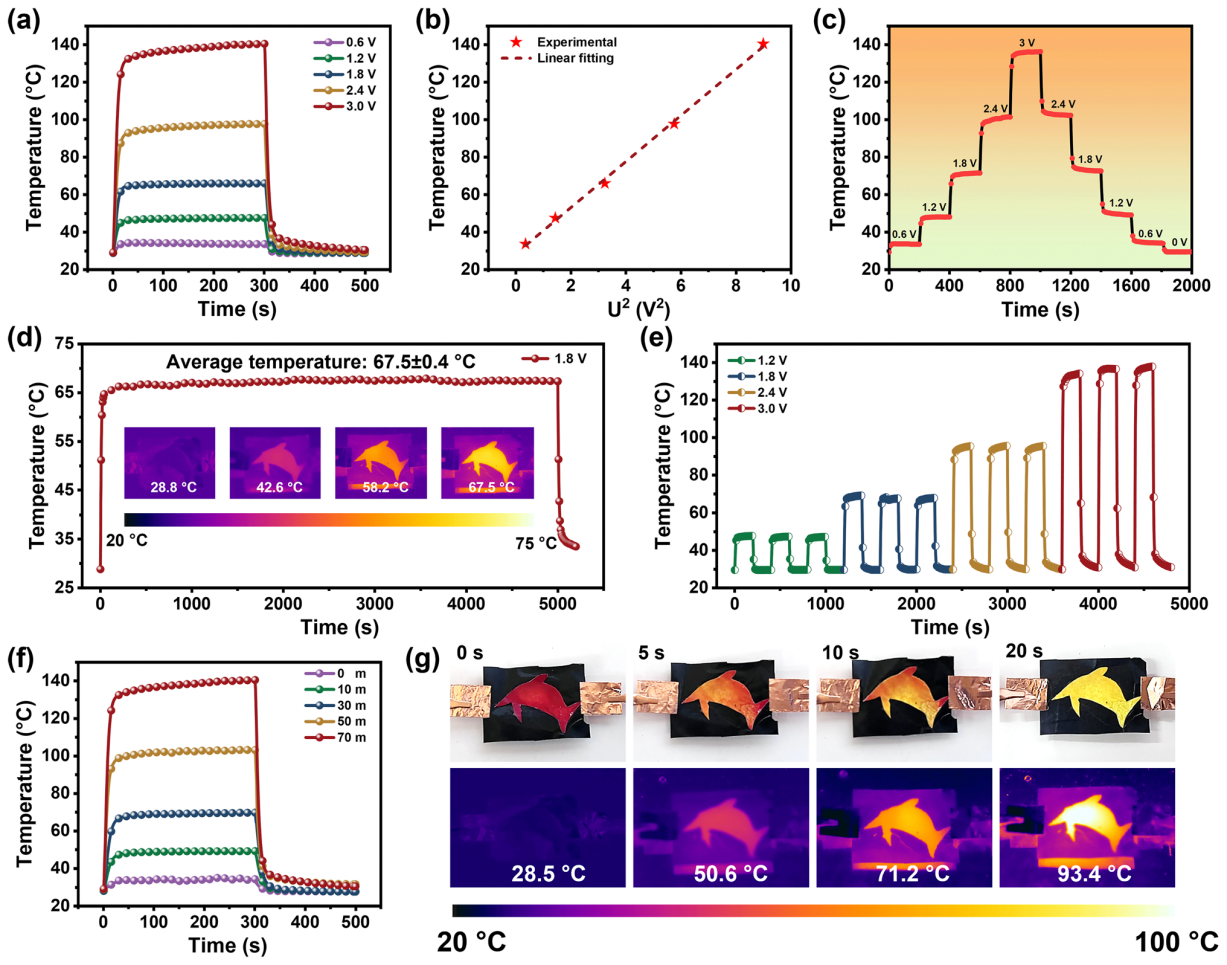
Joule heating capacity of FCFe/M membranes. **a** Temperature variation curves of the FCFe/M-70 m membrane at different voltages. **b** Experimental data and fitting linear of saturation temperature versus U^2^. **c** Variations curve of surface temperature of the FCFe/M-70 m membrane under a 0.6 V voltage fluctuation. **d** Long-term heating stability tests at a voltage of 3 V for FCFe/M-70 m. **e** Cyclic stability experiments of the FCFe/M-70 m membrane. **f** Surface temperature–time curves of FCFe/M membranes with different MXene contents under a 3 V voltage. **g** Visualization of the Joule heating performance of the FCFe/M-70 m membrane with different heating times at 2.4 V, along with their corresponding infrared images

The outstanding electrical and thermal conductivity of the flexible FCFe/M membranes render them strong candidates for use in portable electric heaters. To this end, a systematic evaluation of their Joule heating properties was conducted (Fig. 6). A high correlation coefficient (0.9928) for the linear *I–V* fitting curve demonstrates that the electrothermal behavior of the composite film adheres closely to ohmic law, confirming its reliability as an efficient electrothermal heater (Fig. S11). Figure 6a illustrates the temperature variation curves of the FCFe/M-70 m membrane under different applied voltages. Upon the application of voltage, the surface temperature of the FCFe/M membrane rises rapidly, reaching a saturation temperature (*T*_s_). Upon the removal of the voltage, the FCFe/M membrane cools rapidly, reaching its original state within 20 s. This behavior demonstrates a rapid and reversible Joule heating effect. Notably, at a low voltage of 0.6 V, the *T*_s_ of the FCFe/M membrane stabilizes around 33.6 °C, which approximates the human body’s comfort temperature [66]. As the applied voltage increases, T_s_ progressively rises, achieving 47.5, 65.9, 97.7, and 140.4 °C at voltages of 1.2, 1.8, 2.4, and 3 V, respectively. Additionally, the heating rate of the membrane increases significantly with higher voltages, reaching a temperature rise of over 100 °C within 30 s at 3 V. These results indicate that the FCFe/M membranes exhibit superior electrothermal conversion efficiency and rapid response characteristics.

In essence, the *T*_s_ of an electric heater is determined by the balance between the Joule heat generated and the energy dissipated [67]. This relationship is articulated in Eq. S9, where *T*_s_ is primarily governed by factors such as electrical resistance, surface area, and the applied voltage. Furthermore, as outlined in Eq. S10, *T*_s_ increases linearly in proportion to the square of the supplied voltage (U^2^). As depicted in Fig. 6b, the *T*_s_ of the FCFe/M membrane presents an excellent linear correlation with U^2^, demonstrating their remarkable resistance stability and dimensional integrity. To further assess the temperature controllability of the FCFe/M membrane, real-time temperature changes were monitored across a voltage range of 0.6–3 V. As shown in Fig. 6c, the FCFe/M-70 m membrane achieves rapid temperature regulation under voltage fluctuations as low as 0.6 V, due to the high response power generated by the current, underscoring its significant potential for applications in smart temperature control devices.

Long-term heating stability and cyclic heating/cooling tests were conducted to evaluate the reliability and durability of the FCFe/M membranes as a Joule heating device. As shown in Fig. 6d, the FCFe/M-70 m membrane maintains its equilibrium temperature at 1.8 V for at least 5000 s, with infrared thermal imaging confirming a uniform temperature distribution throughout the heating process. Additionally, the composite membrane exhibits consistent and stable temperature cycling across a voltage range of 1.2–3 V (Fig. 6e). These findings imply that the FCFe/M membrane possesses exceptional performance stability and reusability. It is worth noting that, thanks to the Janus structure of the FCFe/M membrane, with one conductive side and one nearly nonconductive side, the nonconductive FCFe side can safely contact the human body during electrical heating, significantly enhancing its safety in flexible wearable devices. Furthermore, the equilibrium temperature of the FCFe/M-70 m membrane exhibited a gradual increase with the increase in MXene content (Fig. 6f). This phenomenon can be attributed to the high conductivity resulting from the elevated concentration of MXene. Finally, the visual Joule heating behavior of the composite membrane was examined at 2.4 V. As illustrated in Fig. 6g, the FCFe/M membrane displayed a noticeable color change within just 5 s, with the dolphin pattern completely transitioning from red to yellow after 20 s of heating. This rapid and pronounced thermochromic response allows for effective monitoring of environmental temperature changes, offering a valuable tool for detecting potential high-temperature hazards, such as fires.

### Photothermal Conversion Performance of the FCFe/M Membranes

**Fig. 7:**
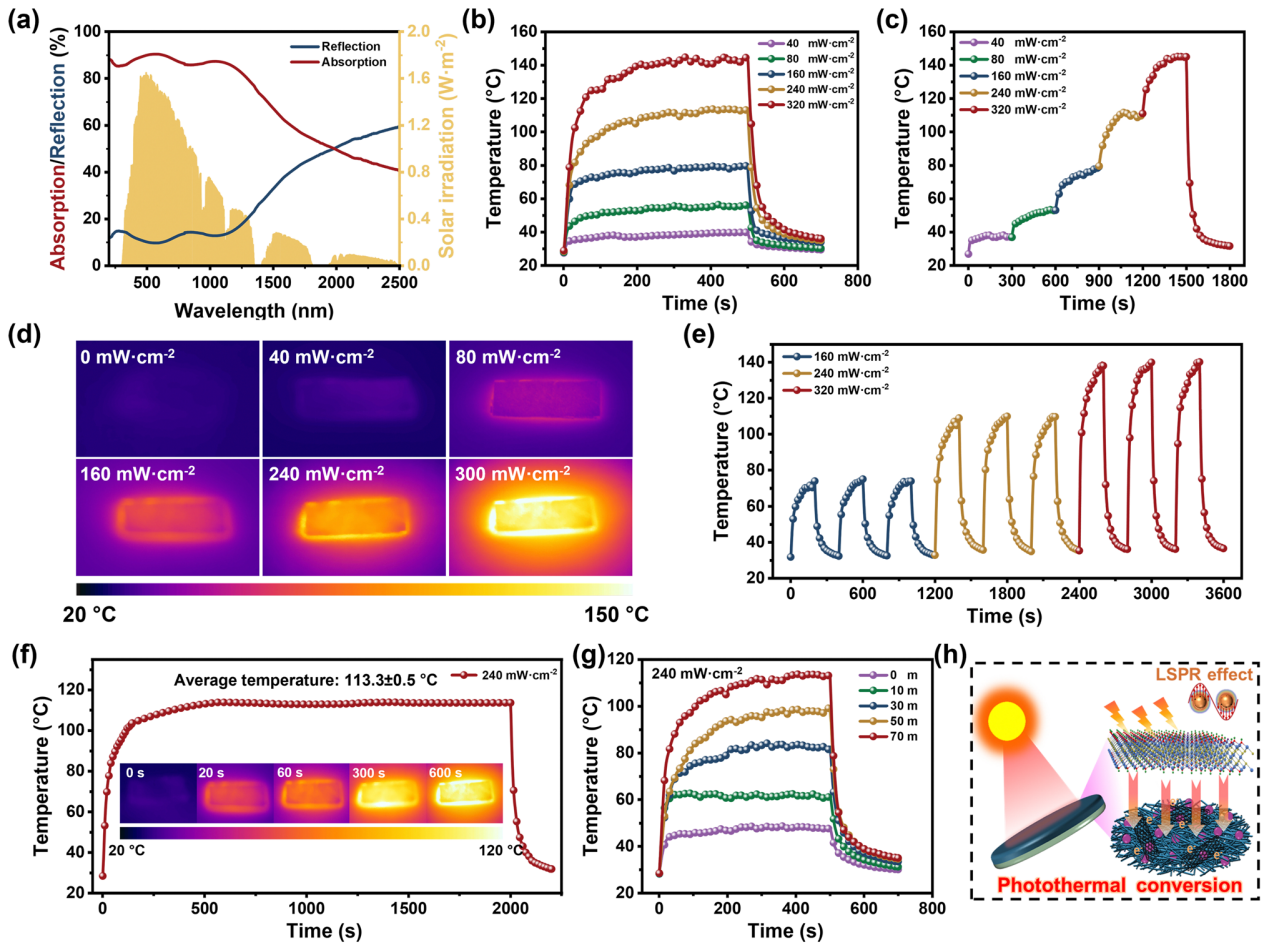
Photothermal conversion performance of FCFe/M membranes. **a** UV–Vis-NIR absorption and reflection spectra of the FCFe/M-70 m membrane. **b** Temperature–time variation curves of the FCFe/M-70 m membrane under simulated sunlight exposure. **c** Surface temperature–time variation curves of the FCFe/M-70 m after changes in optical power density gradients. **d** Infrared images of the FCFe/M-70 m membrane under different optical power densities. **e** Temperature profile of the FCFe/M-70 m membrane at different optical power densities during 3 on/off cycles. **f** Long-term photothermal stability testing of the FCFe/M-70 m. **g** Surface temperature and optical power density curve of FCFe/M membranes with different MXene contents. **h** Photothermal conversion mechanism of FCFe/M Janus membranes

Owing to the photoinduced coherent oscillations of surface electrons in the MXene layer, FCFe/M membranes exhibit exceptional photothermal conversion capabilities [68]. This enables them to directly capture energy from natural sunlight, thereby achieving an effective conversion of light into heat. As shown in Fig. 7a, the UV–Vis-NIR spectra clearly demonstrate the excellent light absorption capability of the FCFe/M-70 m membrane, with solar absorption rates reaching up to 90.34%. This high absorption within the solar spectrum endows FCFe/M Janus membranes with the requisite radiative heating capability. Upon exposure of the MXene side of the FCFe/M-70 m membrane to simulated sunlight, the surface temperature of the composite film exhibited a rapid increase, gradually reaching a steady state (Fig. 7b). Furthermore, as the optical power density is increased from 40 to 320 mW cm^−2^, the steady-state surface temperature of the FCFe/M-70 m membrane rises almost linearly from 39.4 to 145.7 °C (Fig. S12). As illustrated in Fig. 7c, the surface temperature of the FCFe/M-70 m membrane can rapidly respond to changes in optical power density, exhibiting a gradient increase, which underscores their highly controllable photothermal conversion capability. Additionally, following the deactivation of the xenon lamp light source, the FCFe/M-70 m membrane cools rapidly, returning to its original state. Infrared thermal imaging vividly illustrates the surface temperature of the FCFe/M-70 m membrane at steady-state under varying optical power densities (Fig. 7d). The composite membrane demonstrates a uniform temperature distribution during the photothermal process, making it a promising candidate for high-performance wearable smart heaters. The cyclic stability of the FCFe/M photothermal material was subsequently evaluated at different optical power densities (Fig. 7e). During multiple cycles of alternating activation and deactivation of the xenon lamp light source, the FCFe/M-70 m membrane exhibited stable and regular heating and cooling curves, maintaining a relatively constant surface temperature at the same power density. Furthermore, the FCFe/M-70 m membrane was able to sustain its steady-state temperature for at least 2000 s, confirming its remarkable reliability and photothermal stability (Fig. 7f). Further investigation into the influence of MXene content on photothermal conversion performance was conducted. As shown in Figs. 7g and S13, under an optical power density of 240 mW cm^−2^, the steady-state temperature of the FCFe/M membranes exhibits a linear increase with rising MXene content. Additionally, the increase in MXene loading significantly enhances the heating rate of the Janus membranes, indicating that the incorporation of MXene strengthens the photothermal conversion capability.

Moreover, the schematic diagram of the photothermal conversion mechanism of the FCFe/M membranes is illustrated in Fig. 7h. When a laser irradiates the surface of the FCFe/M-M, the MXene layer, owing to its exceptional electrical conductivity and unique layered structure, absorbs a significant amount of photons, causing internal electrons to transition to higher energy levels. These excited electrons subsequently interact with phonons, rapidly releasing energy and thereby generating heat. Furthermore, the presence of lattice atoms in MXene enhances light absorption and photothermal conversion efficiency through the localized surface plasmon resonance (LSPR) effect [69, 70]. When the residual energy penetrates the MXene layer and reaches the FCFe layer, the conjugation and hyperconjugation effects in CNTs enable strong absorption in the near-infrared (NIR) region and enhance electron mobility. Electrons in the π orbital are excited to the π* orbital upon photon absorption and release heat as they return to the ground state [71]. The incident laser undergoes multiple energy conversions inside the film due to the synergistic effects of MXene and CNT, resulting in rapid and efficient photothermal conversion. Based on the discussion above, we successfully fabricated a flexible Janus-structured material through a simple and novel shear-induced in situ fibrillation method. This material exhibits excellent multifunctional properties, including superior EMI shielding performance, weather resistance, thermal conductivity, and multisource-driven self-heating capabilities. Furthermore, the comprehensive property of this multifunctional membrane was compared with previously reported EMI shielding materials, and the FCFe/M Janus membrane achieves more efficient EMW absorption and superior electrothermal-photothermal performance at a relatively small thickness [16, 35, 72–74].

## Conclusion

In summary, a flexible multifunctional FCFe/M composite membrane with a Janus structure were prepared by a combination of shear-induced in situ fibrillation and vacuum-assisted filtration process for EMI shielding and thermal management. The highly conductive MXene layer was tightly bonded to the flexible and robust silk-like FCFe nanofibrous network through hydrogen bonds, which endowed the composite film with outstanding electrical/thermal conductivity, mechanical properties, thermal stability, superhydrophobicity, and flame retardancy. Moreover, the strategic distribution of the conductive reflective layer and the magnetic absorption layer enables the Janus membranes to effectively attenuate EMWs through an absorption-reflection-reabsorption mechanism when the waves enter from the FCFe layer. Notably, the FCFe/M membrane with a thickness of only 84.9 µm achieved a remarkable EMI SE of 44.56 dB and the SSE value reached up to 10,421.3 dB cm^2^ g^−1^ in the X-band, surpassing many conventional shielding materials. Simultaneously, leveraging the excellent electrical and thermal conductivity of MXene and the Janus structure, the FCFe/M membrane demonstrates outstanding performance in personalized thermal management applications. The FCFe/M membrane achieves in-plane and through-plane thermal conductivities of 19.89 and 1.92 W m^−1^ K^−1^, respectively, with an anisotropy ratio of 10.35. Furthermore, the FCFe/M membrane exhibits excellent electrothermal and photothermal conversion capabilities. Under appropriate electrical stimulation (0.6–3 V) and light irradiation (40–320 mW cm^−2^), the FCFe/M heating material achieves maximum surface temperatures of 140.4 and 145.7 °C, respectively, demonstrating rapid, stable, and efficient heating and dissipation processes. These characteristics render FCFe/M membranes a promising candidate for self-heating applications in cold regions. Hence, the innovative Janus structure design proposed in this study provides a practical solution for developing EMI shielding materials with both robust shielding and effective antireflection properties. With their exceptional flexibility, hydrophobicity, flame retardancy, reliability, and thermal management performance, FCFe/M Janus membranes hold significant application potential in aerospace, military, artificial intelligence, smart heating devices, and next-generation flexible wearable electronics.

## Supplementary Information

Below is the link to the electronic supplementary material.

Supplementary file1 (MP4 24316 kb)

Supplementary file2 (DOCX 2641 kb)
