# Supplementary material for: Multifunctional Janus-Structured Polytetrafluoroethylene-Carbon Nanotube-Fe3O4/MXene Membranes for Enhanced EMI Shielding and Thermal Management
Source: Nanomicro Lett. 2025 Feb 6;17:136. doi: 10.1007/s40820-025-01647-x (PMC11802968; doi:10.1007/s40820-025-01647-x)
Supplement: Supplementary file 2 — Supplementary file2 (DOCX 2641 kb) [file 40820_2025_1647_MOESM2_ESM.docx]

Supporting Information for

**Multifunctional Janus-Structured** **Polytetrafluoroethylene-Carbon Nanotube-Fe_3_O_4_/MXene Membranes for Enhanced EMI Shielding and Thermal Management**

Runze Shao^1^, Guilong Wang^1,^ *, Jialong Chai^1^, Jun lin^1^, Guoqun Zhao^1^, Zhihui Zeng^1^, Guizhen Wang^2^

^1^ Key Laboratory for Liquid-Solid Structural Evolution and Processing of Materials (Ministry of Education), Shandong University, Jinan, Shandong 250061, P. R. China

^2^ Key Laboratory of Chinese Education Ministry for Tropical Biological Resources, Hainan University, Haikou, Hainan 570228, P. R. China

*Corresponding author. E-mail: [guilong@sdu.edu.cn](mailto:guilong@sdu.edu.cn) (Guilong Wang)

**S1 Simulation Settings and Parameters**

The electromagnetic simulation was conducted using CST STUDIO SUITE 2023. The model dimensions were set to a length of 22.86 mm, a width of 10.16 mm, and a thickness of 140 mm. The surrounding space parameters were defined with lower and upper X and Y distances of 0, and lower and upper Z distances of 80 mm. Boundary conditions were applied with electric field (E_t_=0) constraints at X_min_, X_max_, Y_min_, and Y_max_, while Z_min_ and Z_max_ were set to "open (add space)" conditions. The simulation frequency range was specified from 8.2 GHz to 12.4 GHz. Waveguide ports were configured with Port 1 aligned along the Z-axis with a negative orientation, and Port 2 aligned along the Z-axis with a positive orientation, both spanning the full plane. Field monitors were set at a frequency of 10.3 GHz to capture the electric field, magnetic field, power, and surface current. The frequency domain solver employed a broadband sweep with a general-purpose tetrahedral mesh, and the excitation source was set to Port 1, with all modes enabled.

The Janus film model was placed centrally within the waveguide cavity, with the EM wave-absorbing layer (FCFe) facing Port 1 and the EM wave-reflecting layer (MXene) facing Port 2. The absorbed layer had a thickness of 59.2 µm, while the reflective layer had a thickness of 25.7 µm. Material parameters for all components were defined using raw data sets for different samples or the material parameter library provided within the software.

**S2 Theoretical Calculation of EMI Shielding Performance**

The Transfer Matrix Method is employed to calculate the complex reflection coefficient (*R*) and transmission coefficient (*T*) of a homogeneous shielding sample. The electric and magnetic fields of a time harmonic (e^j^*^ωt^*) plane wave, propagating perpendicular to the shields, at the incident face should satisfy the continuity conditions. The continuity of the tangential parts of both fields at the incident face of shields generate the boundary conditions:

$\left\{ \begin{aligned} A_{0i}e^{-ik_{0}z_{0}}+B_{0i}e^{ik_{0}z_{0}}=A_{1}e^{-ik_{1}z_{0}}+B_{1}e^{ik_{1}z_{0}} \\ Y_{0}\left( A_{0i}e^{-ik_{0}z_{0}}-B_{0i}e^{ik_{0}z_{0}} \right)=Y_{1}\left( A_{1}e^{-ik_{1}z_{0}}-B_{1}e^{ik_{1}z_{0}} \right) \end{aligned} \right.$ (S1)

where A and B are the coefficients of forward-travelling and backward-travelling waves, $k=\sqrt{\mu\varepsilon}$ is the wave number, $Y=\sqrt{\varepsilon/\mu}$ is the admittance of shields, *μ* and *ε* are the complex permeability and permittivity of shields, the subscripts 0 and 1 are variables relating to the air and the shields, respectively. In terms of the nonmagnetic shields, equals to 1. Moreover, the complex permittivity is composed of the real part and imaginary part:

$\varepsilon=\varepsilon^{'}-j\varepsilon"=\varepsilon^{'}(1-j\frac{\sigma}{\omega\varepsilon^{'}})$ (2)

where w is the angular frequency and σ is the conductivity of shields. Here, we set the real part (*ε* ') equal to the with *ε*_0_, leading to a conductivity-caused EMI shielding calculation of the homogenous shields. At the wave emergent face of the shields, the boundary condition gives:

$\left\{ \begin{aligned} A_{1}e^{-ik_{1}z_{1}}+B_{1}e^{ik_{1}z_{1}}=A_{0t}e^{-ik_{0}z_{1}} \\ Y_{1}\left( A_{1}e^{-ik_{1}z_{1}}-B_{1}e^{ik_{1}z_{1}} \right)=Y_{0}A_{0t}e^{-ik_{0}z_{1}} \end{aligned} \right.$ (3)

Therefore, the complex reflection coefficient *R* and transmission coefficient *T* of the shields can be calculated as：

$R=\frac{B_{0r}}{A_{0i}}$ (4)

$T=\frac{A_{0t}}{A_{0i}}$ (5)

Furthermore, the *SE_T_* and *SE_R_* of the shields in dB can be calculated:

${SE}_{T}=10\log\frac{1}{T^{2}}$ (6)

${SE}_{R}=10\log\frac{1}{{1-R}^{2}}$ (7)

**S3 Calculations of Joule Heating Effect**

The Joule heating effect of FCFe/M membranes can be analyzed using the following equation:

$\boldsymbol{Q=}\frac{\boldsymbol{U}^{\boldsymbol{2}}}{\boldsymbol{R}}\boldsymbol{t}$ (8)

where *Q* is the generated Joule heat, *U* stands for the supplied voltage, *R* is the resistance, *t* represents the working time [S1]. In essence, the surface temperature was a product of the equilibrium between the generated Joule heat and the dissipated power [S2]. The calculation of FCFe/M surface temperature (T_s_) can be achieved through the following equation:

$\boldsymbol{T}_{\boldsymbol{s}}\boldsymbol{=}\boldsymbol{T}_{\boldsymbol{0}}\boldsymbol{+}\frac{\boldsymbol{Q}}{\boldsymbol{Ah}}$ (9)

Where *T_0_* represents the environment temperature, A is the surface area of electrical heater, and h stands for the sum of the heat transfer coefficient. Combined with Equation S8, this Equation S9 can be rewritten as

$\boldsymbol{T}_{\boldsymbol{s}}\boldsymbol{=}\boldsymbol{T}_{\boldsymbol{0}}\boldsymbol{+}\frac{\boldsymbol{U}^{\boldsymbol{2}}}{\boldsymbol{RAh}}$ (10)

S4 Supplementary Figures and Tables


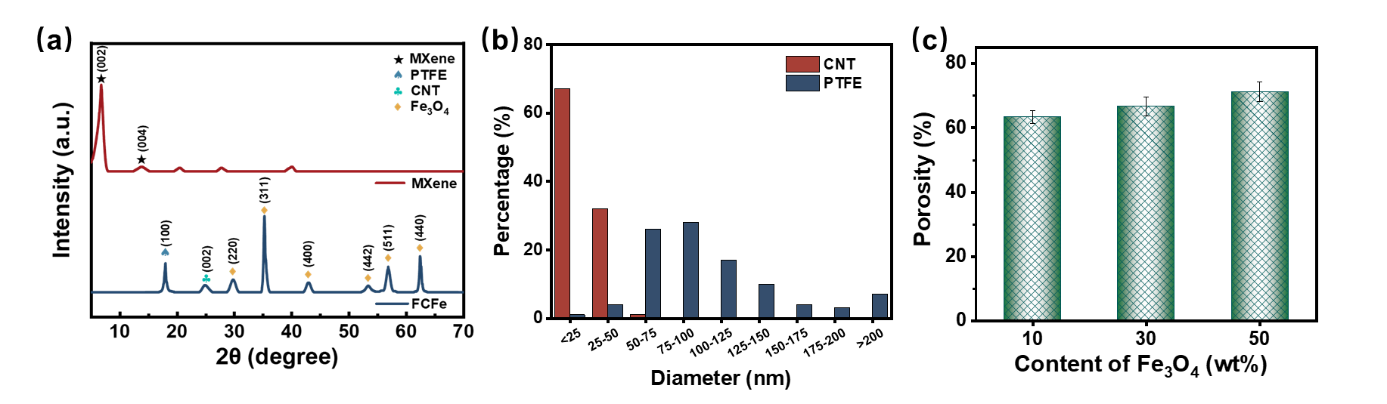


**Fig. S1** (**a**) The XRD patterns of MXene and FCFe. (**b**) The porosity of the FCFe membranes with different content of Fe_3_O_4_


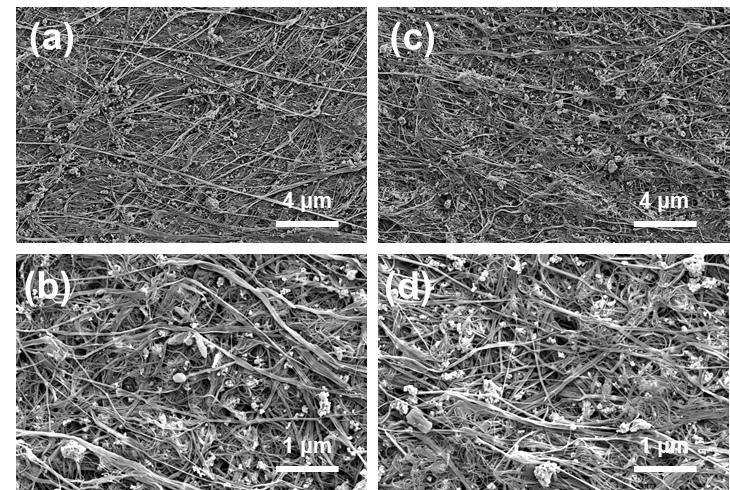


**Fig. S2** SEM images and their enlarged images of FCFe membranes with different content of Fe_3_O_4_. (**a, b**) FCFe membrane with 10 wt% Fe_3_O_4_. (**c, d**) FCFe membrane with 30 wt% Fe_3_O_4_


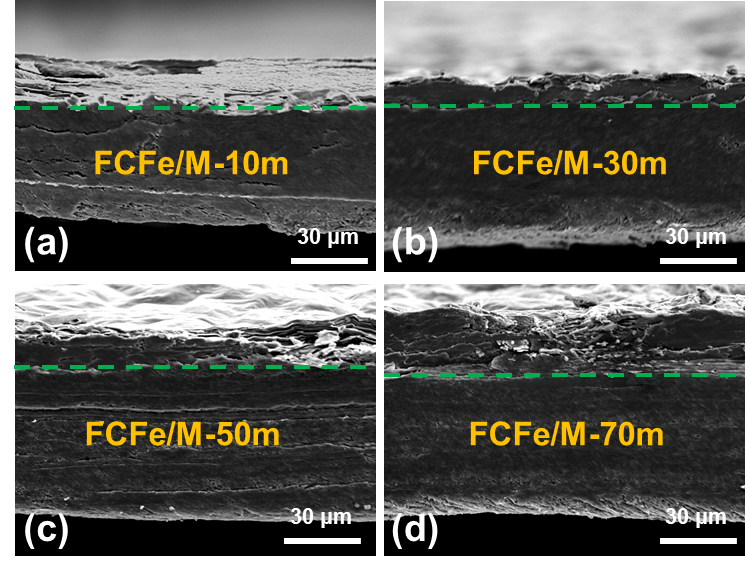


**Fig. S3** SEM image of the cross section of (**a**) FCFe/M-10m, (**b**) FCFe/M-30m, (**c**) FCFe/M-50m, and (**d**) FCFe/M-70m


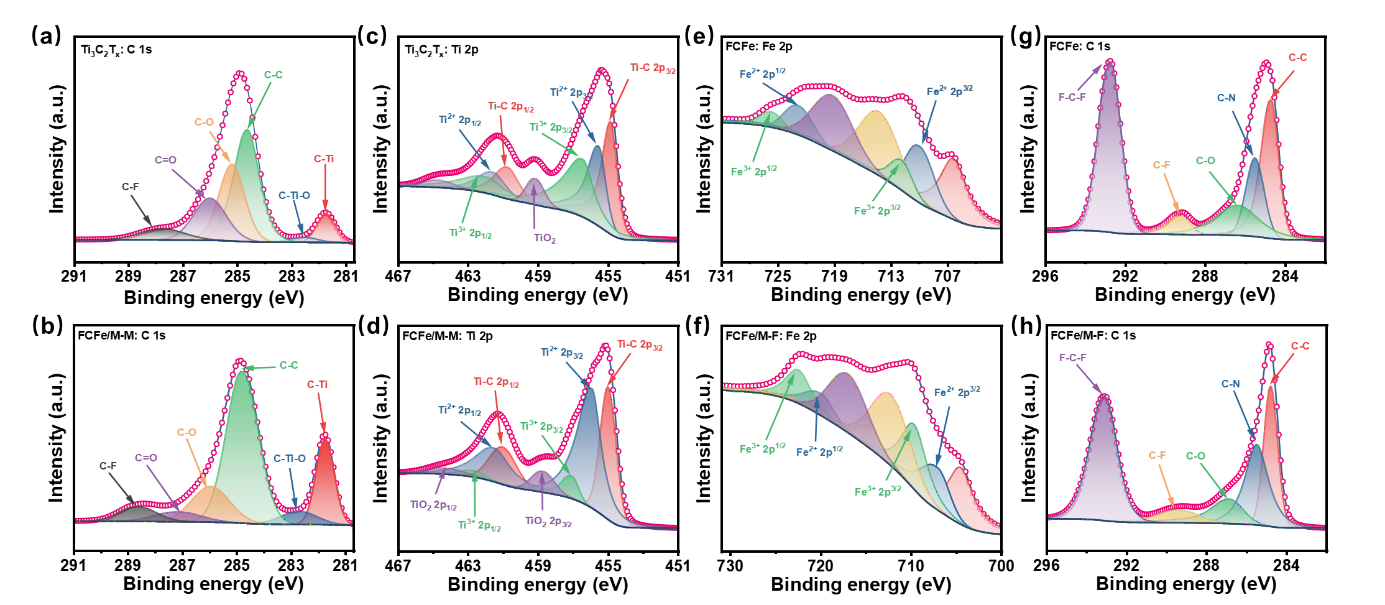


**Fig. S4** (**a, b**) C 1s spectra of MXene and FCFe/M-F. (**c, d**) Ti 2p spectra of MXene and FCFe/M-M. (**e, f**) Fe 2p spectra of FCFe and FCFe/M-F. (**g, h**) C 1s spectra of FCFe and FCFe/M-M


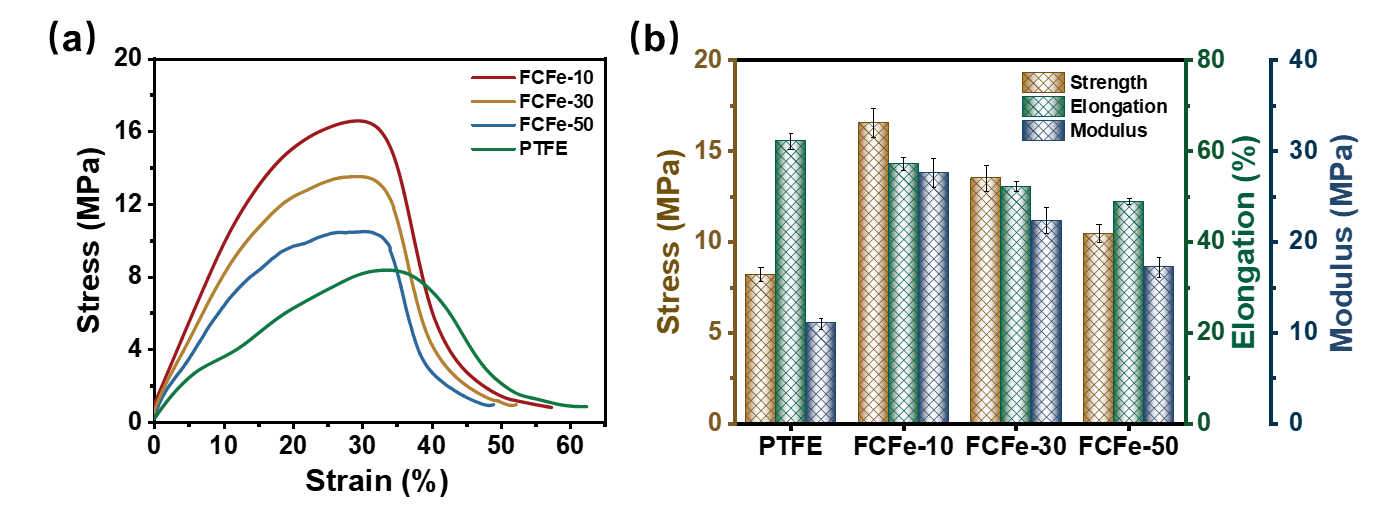


**Fig. S5** (**a**) Stress-strain curves of PTFE film and FCFe membranes. (**b**) Statistics of tensile stress, elongation at break, and Young's modulus of PTFE film and FCFe membranes


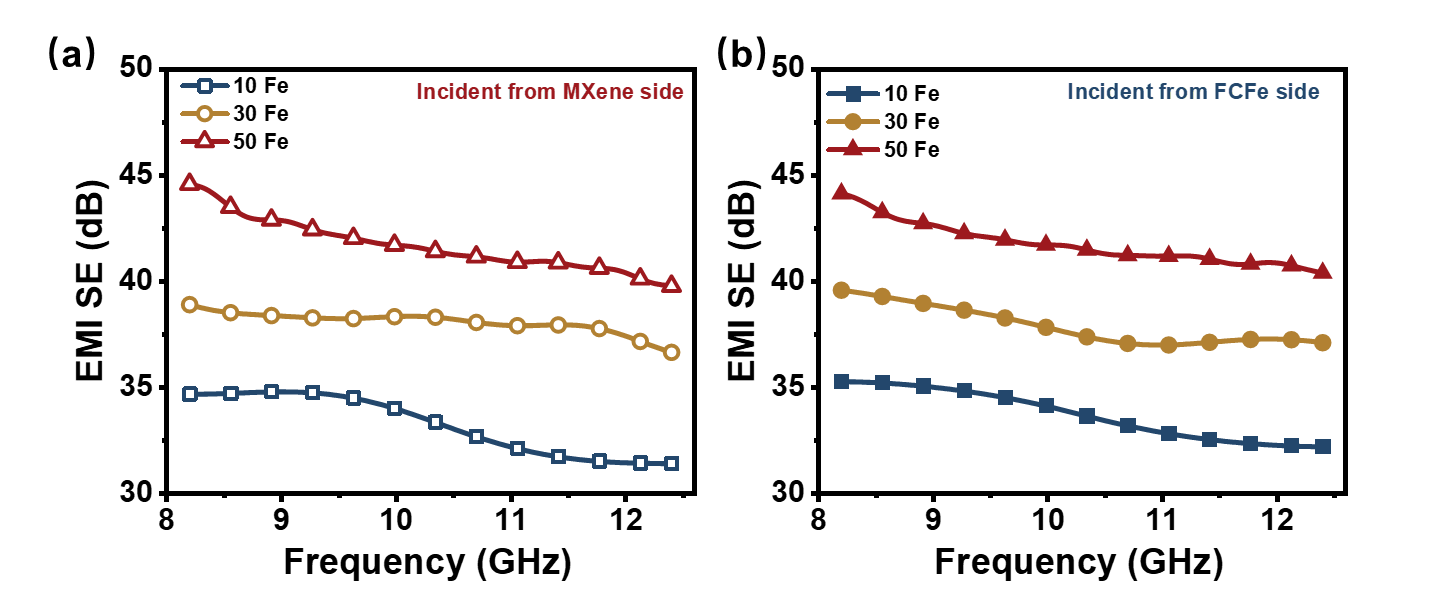


Fig. S6 The EMI SE of FCFe/M membranes with different content of Fe_3_O_4_ when the EMWs incident from (a) MXene side and (b) FCFe side


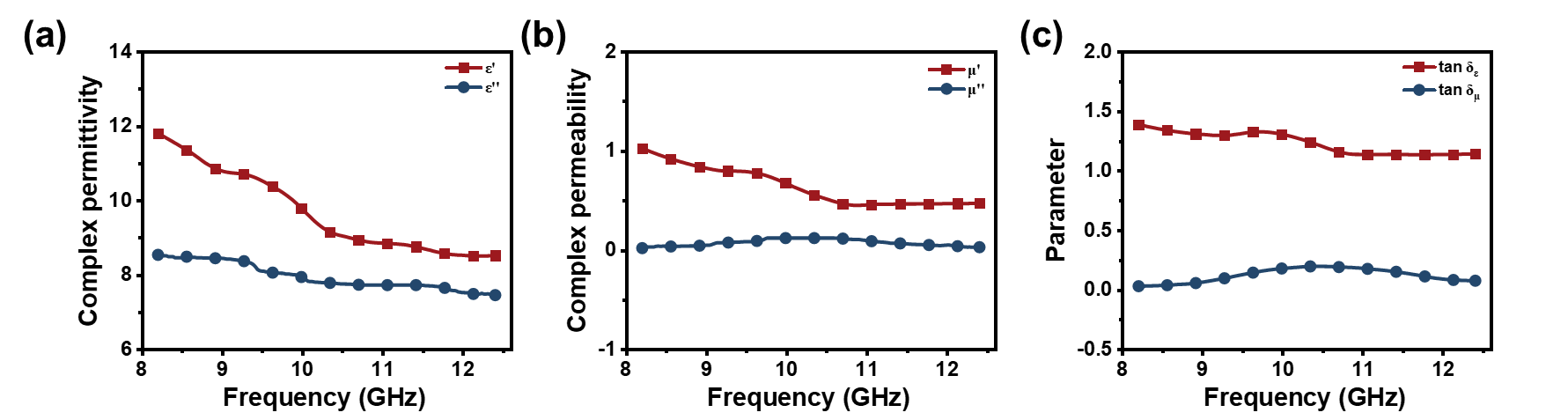


**Fig. S7** (**a**) complex permittivity, (**b**) complex permeability, and (**c**) tangential loss of the FCFe membrane


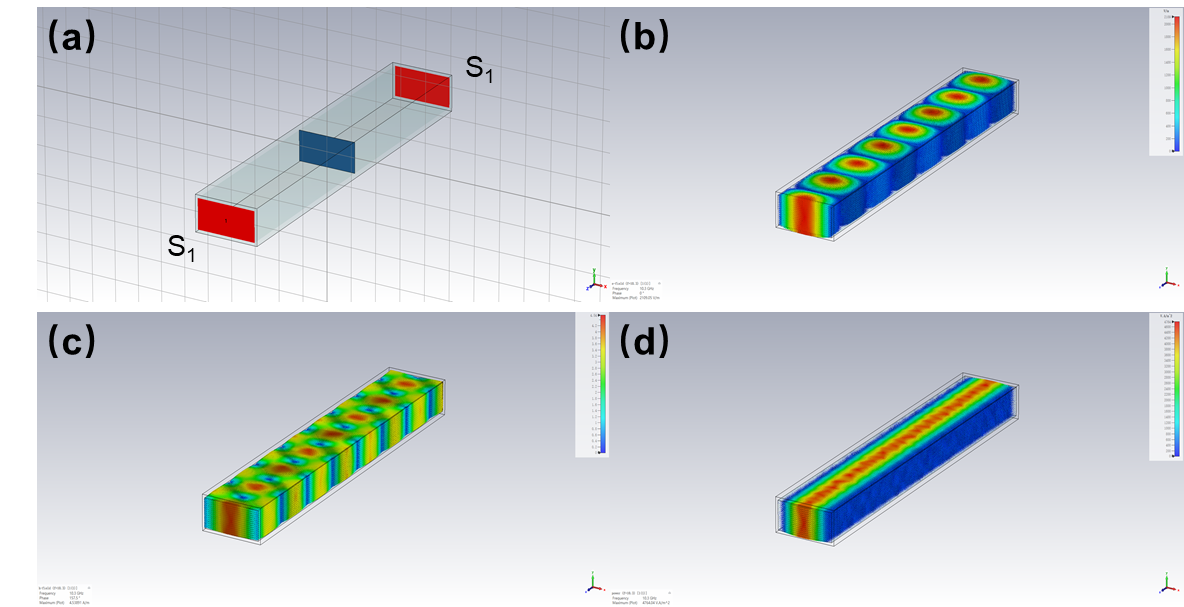


Fig. S8 (a) Schematic of modeling in CST simulation. Simulation diagram of (b) electric field distribution, (c) magnetic field distribution, and (d) power flow in the rectangular waveguide clamp


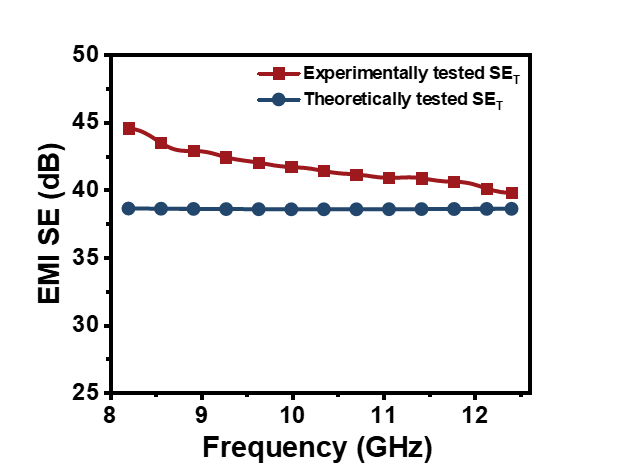


**Fig. S9** Experimentally tested EMI SE and theoretically calculated EMI SE of the FCFe/M-70m membrane

**
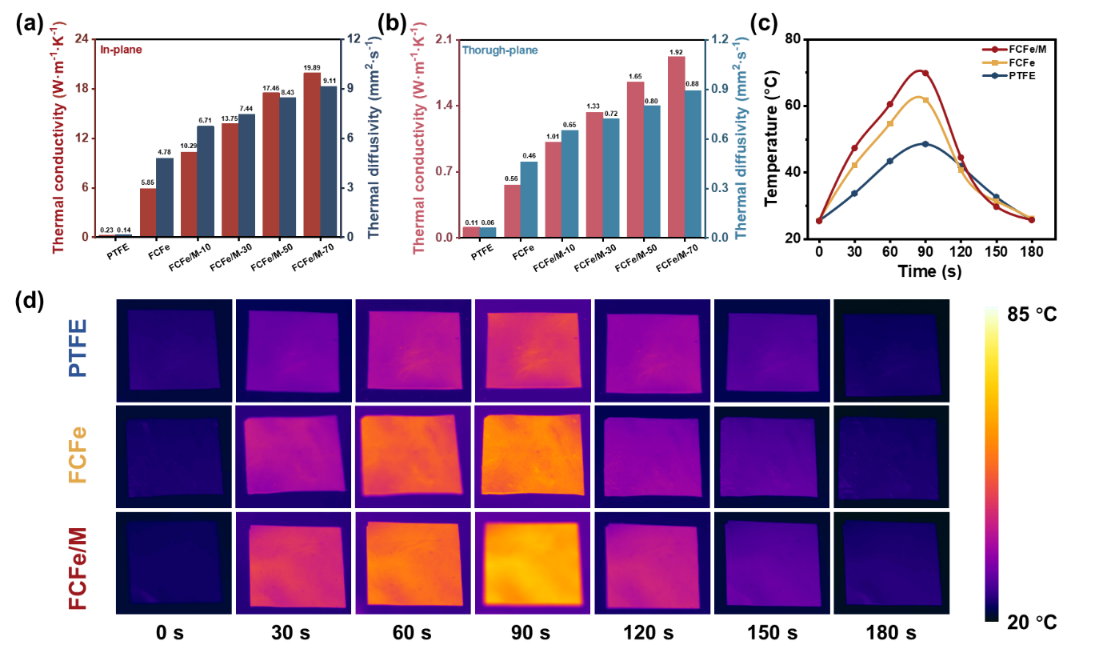
**

**Fig. S10** (a) The thermal conductivity and thermal diffusivity of PTFE, FCFe, and FCFe/M membranes. (b) The surface temperature curve of PTFE, FCFe, and FCFe/M membranes. (c) Thermal imaging spectra of composite film heating to cooling

**Fig. S11** *I-V* curves of the FCFe/M-70m membrane

**Fig. S12** Steady-state surface temperature and optical power density of the FCFe/M-70m membranes

**Fig. S13** Steady-state surface temperature and content of MXene curve of the FCFe/M membranes

**
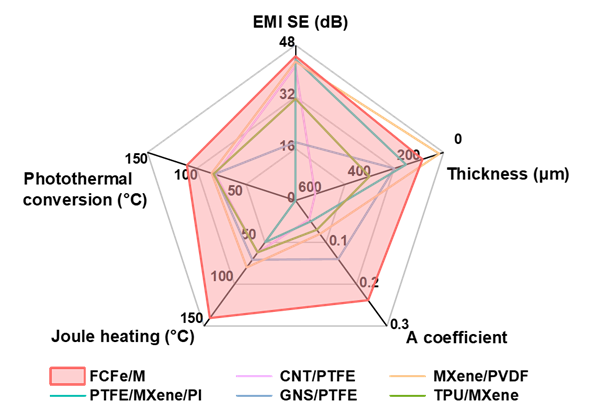
**

**Fig. S14** Comprehensive performance comparison between the FCFe/M membranes and samples reported in the literature

**Table S1** The conductivity and saturation magnetization values of the FCFe membranes

| Points | 1 | 2 | 3 | 4 | 5 | 6 |
| --- | --- | --- | --- | --- | --- | --- |
| Conductivity (S·cm^−1^) | 2.12 | 2.01 | 2.21 | 2.09 | 2.13 | 2.16 |
| Saturation magnetization values (emu·g^−1^) | 42.45 | 41.72 | 43.29 | 44.07 | 40.82 | 42.69 |

**Table S2** Mechanical properties of FCFe/M membranes

| Sample | Tensile strength  (MPa) | Elongation at break  (%) | Young’s modulus (MPa) |
| --- | --- | --- | --- |
| PTFE | 8.21 | 62.22 | 11.03 |
| FCFe-10 | 16.58 | 57.23 | 27.58 |
| FCFe -30 | 13.51 | 52.14 | 22.36 |
| FCFe-50 | 10.47 | 48.88 | 17.25 |
| FCFe/M-10m | 17.11 | 54.89 | 41.47 |
| FCFe/M-30m | 21.31 | 58.80 | 47.16 |
| FCFe/M-50m | 24.46 | 62.06 | 59.46 |
| FCFe/M-70m | 28.14 | 65.86 | 78.95 |

**Table S3** EMI shielding performance of various shielding materials

| Materials | Thickness (mm) | EMI SE  (dB) | SSE  (dB·cm^2^·g^−1^) | Refs. |
| --- | --- | --- | --- | --- |
| FCFe/M-10m | 0.0636 | 22.1 | 2445.3 | This work |
| FCFe/M-30m | 0.0704 | 28.78 | 5310.1 | This work |
| FCFe/M-50m | 0.0783 | 34.42 | 8274.2 | This work |
| **FCFe/M-70m** | **0.0849** | **44.56** | **10421.3** | **This work** |
| MXene/CNF | 0.047-0.167 | 23.5-25.8 | 1326-2647 | [S3] |
| MXene/TONCF | 0.047 | 32.7 | 4761 | [S4] |
| CNF@MXene | 0.035 | 40 | 7029 | [S5] |
| MXene/PNFs | 0.028 | 43 | 8399 | [S6] |
| MXene/PVA | 0.1-0.3 | 26-28 | 3867-4770 | [S7] |
| MXene/MMT | 0.033-0.045 | 28-46 | 3254-6336 | [S8] |
| MXene/PS | 2 | 62 | 255.2 | [S9] |
| MXene/PVA aerogel | 10.8 | 28 | 2586 | [S10] |
| MWCNT/cellulose aerogel | 2.5 | 20-35 | 1700-3776 | [S11] |
| CNT/PMMA | 4.5 | 30 | 49 | [S12] |
| CNT/WPU | 0.32 | 35 | 779 | [S13] |
| CNT/WPU foam | 2.3 | 50.5 | 1743 | [S14] |
| Graphene/WPU | 2 | 32 | 153 | [S15] |
| Flexible Graphite | 0.2 | 110 | 500 | [S16] |
| Graphene/PMMA | 3.4 | 30 | 74 | [S17] |
| Graphene/PEI | 2.3 | 40 | 68 | [S18] |
| Copper | 3.1 | 90 | 32 | [S19] |
| Cu NW/PS | 0.21 | 35 | 158.7 | [S20] |

**Supplementary References**

1. J. Wang, X. Ma, J. Zhou, F. Du, C. Teng, Bioinspired, high-strength, and flexible mxene/aramid fiber for electromagnetic interference shielding papers with joule heating performance. ACS Nano **16**(4), 6700-6711 (2022). https://doi.org/10.1021/acsnano.2c01323
2. Y. Cheng, H. Zhang, R. Wang, X. Wang, H. Zhai et al., Highly stretchable and conductive copper nanowire based fibers with hierarchical structure for wearable heaters. ACS Appl. Mater. Interfaces **8**(48), 32925-32933 (2016). https://doi.org/10.1021/acsami.6b09293
3. W.-T. Cao, F.-F. Chen, Y.-J. Zhu, Y.-G. Zhang, Y.-Y. Jiang et al., Binary strengthening and toughening of mxene/cellulose nanofiber composite paper with nacre-inspired structure and superior electromagnetic interference shielding properties. ACS Nano **12**(5), 4583-4593 (2018). https://doi.org/10.1021/acsnano.8b00997
4. Z. Zhan, Q. Song, Z. Zhou, C. Lu, Ultrastrong and conductive mxene/cellulose nanofiber films enhanced by hierarchical nano-architecture and interfacial interaction for flexible electromagnetic interference shielding. J. Mater. Chem. C **7**(32), 9820-9829 (2019). https://doi.org/10.1039/c9tc03309b
5. B. Zhou, Z. Zhang, Y. Li, G. Han, Y. Feng et al., Flexible, robust, and multifunctional electromagnetic interference shielding film with alternating cellulose nanofiber and mxene layers. ACS Appl. Mater. Interfaces **12**(4), 4895-4905 (2020). https://doi.org/10.1021/acsami.9b19768
6. L. Wang, Z. Ma, Y. Zhang, H. Qiu, K. Ruan et al., Mechanically strong and folding‐endurance Ti_3_C_2_T_x_ mxene/pbo nanofiber films for efficient electromagnetic interference shielding and thermal management. Carbon Energy **4**(2), 200-210 (2022). https://doi.org/10.1002/cey2.174
7. H. Xu, X. Yin, X. Li, M. Li, S. Liang et al., Lightweight Ti_2_CT_x_ mxene/poly(vinyl alcohol) composite foams for electromagnetic wave shielding with absorption-dominated feature. ACS Appl. Mater. Interfaces **11**(10), 10198-10207 (2019). https://doi.org/10.1021/acsami.8b21671
8. L. Li, Y. Cao, X. Liu, J. Wang, Y. Yang et al., Multifunctional mxene-based fireproof electromagnetic shielding films with exceptional anisotropic heat dissipation capability and joule heating performance. ACS Appl. Mater. Interfaces **12**(24), 27350-27360 (2020). https://doi.org/10.1021/acsami.0c05692
9. R. Sun, H.B. Zhang, J. Liu, X. Xie, R. Yang et al., Highly conductive transition metal carbide/carbonitride(mxene)@polystyrene nanocomposites fabricated by electrostatic assembly for highly efficient electromagnetic interference shielding. Adv. Funct. Mater. **27**(45), 1702807 (2017). https://doi.org/10.1002/adfm.201702807
10. M. Han, X. Yin, H. Wu, Z. Hou, C. Song et al., Ti_3_C_2_ mxenes with modified surface for high-performance electromagnetic absorption and shielding in the x-band. ACS Appl. Mater. Interfaces **8**(32), 21011-21019 (2016). https://doi.org/10.1021/acsami.6b06455
11. L.-Q. Zhang, S.-G. Yang, L. Li, B. Yang, H.-D. Huang et al., Ultralight cellulose porous composites with manipulated porous structure and carbon nanotube distribution for promising electromagnetic interference shielding. ACS Appl. Mater. Interfaces **10**(46), 40156-40167 (2018). https://doi.org/10.1021/acsami.8b14738
12. N.C. Das, Y. Liu, K. Yang, W. Peng, S. Maiti et al., Single‐walled carbon nanotube/poly(methyl methacrylate) composites for electromagnetic interference shielding. Polym. Eng. Sci. **49**(8), 1627-1634 (2009). https://doi.org/10.1002/pen.21384
13. Z. Zeng, M. Chen, H. Jin, W. Li, X. Xue et al., Thin and flexible multi-walled carbon nanotube/waterborne polyurethane composites with high-performance electromagnetic interference shielding. Carbon **96**, 768-777 (2016). https://doi.org/10.1016/j.carbon.2015.10.004
14. Z. Zeng, H. Jin, M. Chen, W. Li, L. Zhou et al., Lightweight and anisotropic porous mwcnt/wpu composites for ultrahigh performance electromagnetic interference shielding. Adv. Func. Mater. **26**(2), 303-310 (2015). https://doi.org/10.1002/adfm.201503579
15. S.-T. Hsiao, C.-C. M. Ma, H.-W. Tien, W.-H. Liao, Y.-S. Wang et al., Using a non-covalent modification to prepare a high electromagnetic interference shielding performance graphene nanosheet/water-borne polyurethane composite. Carbon **60**, 57-66 (2013). https://doi.org/10.1016/j.carbon.2013.03.056
16. A. A. Eddib, D.D.L. Chung, The importance of the electrical contact between specimen and testing fixture in evaluating the electromagnetic interference shielding effectiveness of carbon materials. Carbon **117**, 427-436 (2017). https://doi.org/10.1016/j.carbon.2017.02.091
17. H.-B. Zhang, W.-G. Zheng, Q. Yan, Z.-G. Jiang, Z.-Z. Yu, The effect of surface chemistry of graphene on rheological and electrical properties of polymethylmethacrylate composites. Carbon **50**(14), 5117-5125 (2012). https://doi.org/10.1016/j.carbon.2012.06.052
18. J. Ling, W. Zhai, W. Feng, B. Shen, J. Zhang et al., Facile preparation of lightweight microcellular polyetherimide/graphene composite foams for electromagnetic interference shielding. ACS Appl. Mater. Interfaces **5**(7), 2677-2684 (2013). <https://doi.org/10.1021/am303289m>
19. X.D Shui, D.L. Chung, Nickel filament polymer-matrix composites with low surface impedance and high electromagnetic interference shielding effectiveness. J. Electron. Mater. **8**(26), 928-934 (1997). https://doi.org/10.1007/s11664-997-0276-4
20. M.H. Al-Saleh, G.A. Gelves, U. Sundararaj, Copper nanowire/polystyrene nanocomposites: Lower percolation threshold and higher emi shielding. Compos. Part A- Appl. S. **42**(1), 92-97 (2011). https://doi.org/10.1016/j.compositesa.2010.10.003
